# Efficacy and safety of laser acupuncture for treating insomnia in major depressive disorder: study protocol for a randomized controlled trial

**DOI:** 10.3389/fpsyt.2025.1698773

**Published:** 2025-11-20

**Authors:** Qiyue Qiu, Mun Fei Yam, Weiwei Yang, Fei Guo, Chenxi Liu, Mohammad Farris Iman Leong Bin Abdullah, Shichang Yang

**Affiliations:** 1 The Second Affiliated Hospital of Xinxiang Medical University, Xinxiang, Henan, China; 2 Department of Pharmacology, School of Pharmaceutical Sciences, Universiti Sains Malaysia, Penang, Malaysia; 3 Department of Psychiatry, The Fifth People’s Hospital of Lingbao City, Lingbao, Henan, China; 4 Department of Psychiatry and Mental Health, Faculty of Medicine, Universiti Sultan Zainal Abidin, Terengganu, Malaysia

**Keywords:** low-dose laser acupuncture, insomnia, major depressive disorder, randomized controlled trial, protocol

## Abstract

**Background:**

Effective options for managing insomnia in major depressive disorder (MDD) remain limited. This randomized, single-blind, three-arm parallel trial will compare low-dose laser acupuncture (LA), sham laser acupuncture (SLA), and standard care, evaluating changes in sleep and mood alongside serum biomarkers at three time points (t0 = pre-treatment; t1 = post-treatment, 6 weeks; t2 = follow-up, 12 weeks after treatment).

**Methods:**

A total of 120 inpatients meeting all eligibility criteria will be randomized (1:1:1) to LA, SLA, or control. The primary outcome is the Pittsburgh Sleep Quality Index (PSQI). Secondary outcomes include the Insomnia Severity Index (ISI), the 17-item Hamilton Depression Rating Scale (HAMD-17), serum 5-hydroxytryptamine (5-HT) and cortisol (CORT), and actigraphy. Outcomes are assessed at t0, t1, and t2. Safety will be evaluated by adverse events (AEs) and discontinuations due to adverse effects. Analyses will follow the intention-to-treat principle.

**Discussion:**

By integrating validated clinical endpoints with biomarker assessments, this trial will provide rigorous evidence on the efficacy and safety of low-dose LA for insomnia comorbid with MDD and help clarify potential mechanisms of action. If effective, LA could offer a non-invasive adjunct or alternative to current therapies.

**Registration Details:**

ClinicalTrials.gov, identifier NCT06443242.

## Introduction

Depression and insomnia are highly prevalent and frequently comorbid conditions that significantly contribute to the global disease burden (1–3). Notably, approximately 70% of individuals with depression experience insomnia—a rate three to four times higher than in the general population (1, 4, 5). Their relationship is bidirectional: insomnia exacerbates depressive symptoms, increases the risk of chronicity and relapse, and elevates suicidal ideation (6–8). Shared pathophysiological mechanisms, including dysregulation of the hypothalamic-pituitary-adrenal (HPA) axis leading to aberrant cortisol (CORT) secretion, and alterations in 5-Hydroxytryptamine (5-HT) neurotransmission, underlie this comorbidity and disrupt sleep architecture (9–11). Effectively managing insomnia is therefore critical to improving overall outcomes in patients with major depressive disorder (MDD).

First-line treatments such as cognitive behavioral therapy for insomnia (CBT-I) face limitations in accessibility and cultural acceptability (7, 8), while pharmacotherapies (e.g., hypnotics, antidepressants) often entail undesirable side effects, risk of dependence, and limited long-term efficacy (9, 10). Traditional acupuncture, which modulates neurotransmitters and inflammatory responses, offers benefits for both sleep and mood disorders (12, 13). However, its needle-based nature causes needling pain, bruising, and rarely, serious adverse events (SAEs), which deter some patients and limit its utility (14–17). These limitations highlight the need for effective, non-invasive alternatives.

Laser acupuncture (LA) is a non-invasive modality that applies low-dose laser stimulation to acupoints, combining principles of traditional acupuncture with modern photobiomodulation (18, 19). It is particularly suitable for needle-sensitive populations (e.g., children, the elderly) and has shown promise in improving sleep and mood symptoms by regulating neuroendocrine function and reducing inflammation (20, 21). Photobiomodulation is thought to exert its effects by stimulating mitochondrial cytochrome c oxidase, thereby enhancing cellular energy metabolism and modulating redox signaling (22–24). These changes may regulate neuroendocrine and immune pathways, contributing to the normalization of 5-HT and CORT levels relevant to sleep–wake regulation (25–27). Nevertheless, robust clinical trials investigating LA for depression-related insomnia are scarce, and its effects on underlying biological mechanisms remain poorly elucidated.

Despite its potential, significant evidence gaps exist regarding LA’s efficacy, mechanisms, and applicability in treating insomnia in MDD patients (28, 29). Few high-quality trials have specifically evaluated LA for this comorbidity, and none have comprehensively assessed its impact on sleep quality alongside biomarker changes (29, 30). Furthermore, the mechanisms by which LA may influence objective sleep outcomes and biochemical markers (e.g., 5-HT, CORT) are not yet established (31, 32). This study aims to address these gaps by evaluating the clinical efficacy and safety of LA in improving sleep quality—assessed via the Pittsburgh Sleep Quality Index (PSQI)—and depressive symptoms in MDD patients, and by exploring its potential effects on serum 5-HT and CORT levels. We hypothesize that LA will be superior to sham laser acupuncture (SLA) and standard care in improving sleep quality, reducing depressive symptoms, and modulating biomarker levels, offering a well-tolerated and non-invasive therapeutic option.

In this trial, we will use a controlled study design (LA group vs. SLA group vs. standard care group) to evaluate the clinical efficacy and safety of LA for insomnia in MDD patients; we will dynamically assess subjective sleep quality (via PSQI) and depressive symptoms at baseline, Week 6 (post-treatment), and 12-week follow-up, compare serum CORT and 5-HT changes among the three groups, evaluate LA’s safety via dropout rates related to adverse events (AEs). We also aim to provide evidence for LA’s advantages over SLA and standard care and offer a non-invasive option for eligible patients if effective.

This study is motivated by the urgent need for effective, non-invasive treatments for insomnia in MDD patients. LA represents a promising alternative because of its non-invasiveness, minimal side effects, and potential suitability for home-based application (18, 19). Confirming its efficacy could provide a valuable treatment option for patients who decline needle-based therapies. By incorporating biomarker assessments, this research also seeks to clarify the biological mechanisms underlying LA’s effects, thereby contributing to a more personalized and mechanism-based approach to treatment. Should LA demonstrate clinical benefits, it could help reduce the disease burden associated with comorbid depression and insomnia, improving functional outcomes and quality of life in affected individuals.

## Methods

### Study design

This is a three-year, single-center, randomized, single-blind, three-arm parallel randomized controlled trial (RCT) conducted at The Second Affiliated Hospital, Xinxiang Medical University (XXMU), China. Ethical approval was obtained from the Ethics Committees of XXMU (XYEFYLL-(Research)-2024-36) and Universiti Sains Malaysia (USM) (USM/JEPeM/PP/24070605). The trial is registered at ClinicalTrials.gov (NCT06443242) and follows Consolidated Standards of Reporting Trials (CONSORT) guidelines.

A total of 120 inpatients with major depressive disorder (MDD) and comorbid insomnia will be randomized (1:1:1) to one of three groups: laser acupuncture (LA)—low-dose laser therapy delivered at acupoints; sham laser acupuncture (SLA)—identical procedures without effective laser output; or standard care.

**Figure 1:**
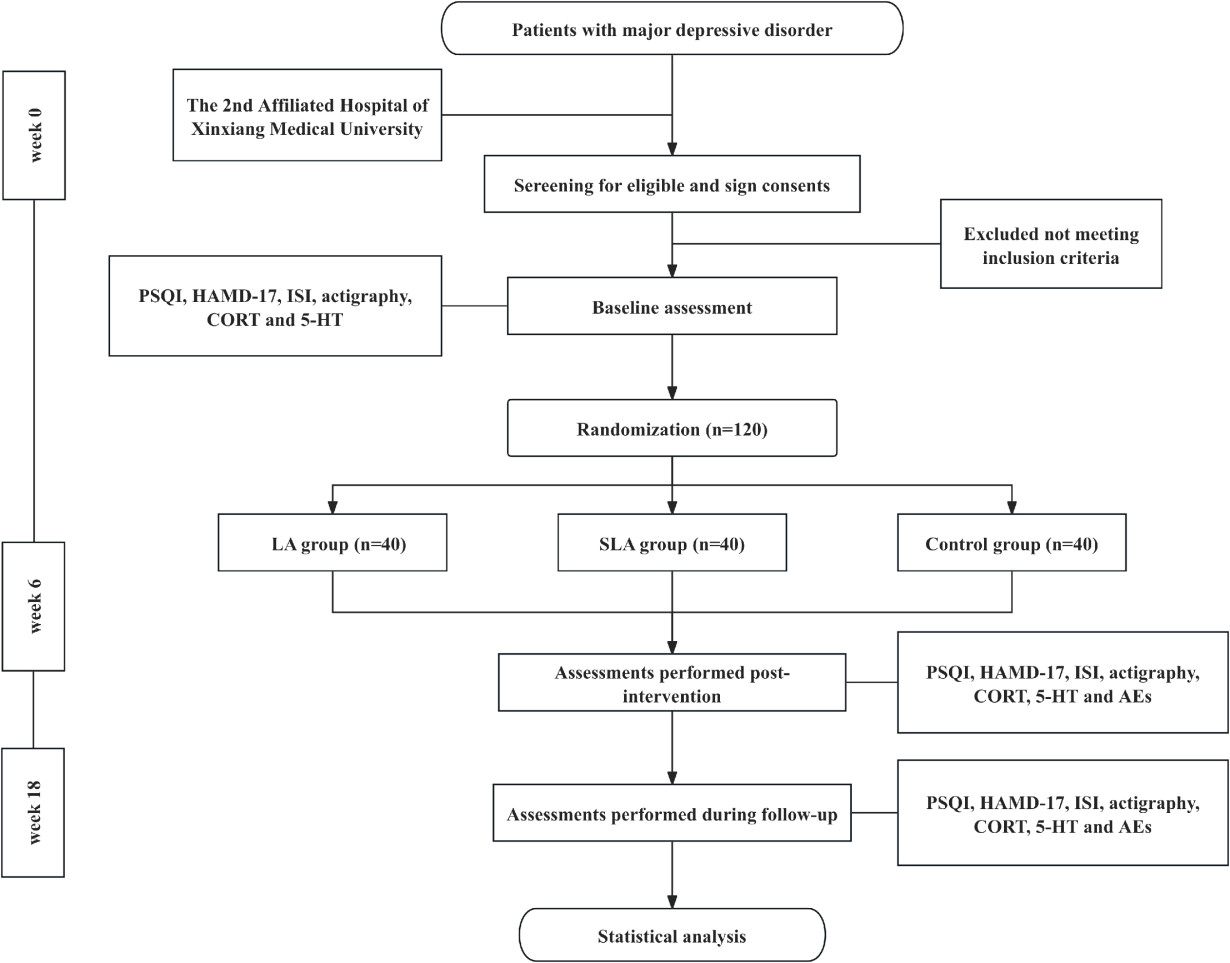
Trial flowchart. LA, laser acupuncture + fixed dose of antidepressant; SLA, sham laser acupuncture + fixed dose of antidepressant; PSQI, Pittsburgh Sleep Quality Index; HAMD-17, 17-item Hamilton Depression Rating Scale; ISI, Insomnia Severity Index; 5-HT, serotonin (5-hydroxytryptamine); AE, adverse events. Each LA and SLA weekly session is characterized by a pre-determined regimen of 5 consecutive days per week, followed by 2 days of rest with 20 minutes per day of laser acupuncture.

The intervention will last 6 weeks with a 12-week follow-up. The primary outcome is sleeping quality (PSQI), while secondary outcomes include depressive symptoms and serum biomarkers (5-HT, CORT). AEs will be monitored and reported to both ethics committees. **Figure 1** provides a flowchart outlining the study procedures and outcome measures. The CONSORT checklist for this RCT is presented in **Supplementary Material 1**, while the CONSORT flow diagram for participant enrolment, allocation, follow up and analysis is presented in **Supplementary Material 2**.

### Patient recruitment

All participants in this trial will be recruited from the Department of Psychiatry, The Second Affiliated Hospital of XXMU, China. Recruitment will be carried out through direct approach by the research team as well as announcements posted on hospital information platforms. Interested patients may contact the study staff using the details provided and will undergo an initial eligibility screening. Those who preliminarily meet the inclusion criteria will then be interviewed face-to-face and assessed at baseline by an independent researcher who is blinded to the study objectives. After screening according to the predefined inclusion and exclusion criteria, eligible patients will be informed that they will be randomized to the LA, SLA, or control group. They will also be clearly informed of the possible benefits and risks of participation. Before enrolment, participants will voluntarily sign a written informed consent, and they will be free to withdraw from the study at any time without providing a reason.

### Inclusion criteria

Inclusion criteria are as follows: (1) inpatients aged 18–60 years with MDD diagnosed by Diagnostic and Statistical Manual of Mental Disorders, Fifth Edition, Text Revision (DSM-5-TR); (2) comorbid insomnia (PSQI >5, HAMD-17 20–35); (3) no hypnotic or acupuncture treatment in the past month; (4) no cognitive or communication disorders; (5) willingness to provide written informed consent and accept randomization; and (6) patients on a stable dose of antidepressants (e.g., escitalopram, venlafaxine, or mirtazapine) for ≥2 weeks prior to enrolment, and agreeing to maintain the same dosage during the trial, will be eligible (33).

### Exclusion criteria

Exclusion criteria are as follows: (1) suicidal tendency; (2) history of psychotic disorder, bipolar disorder, obsessive-compulsive disorder, or post-traumatic stress disorder (PTSD); (3) alcohol or substance use; (4) major medical illness (e.g., liver or kidney dysfunction, tumor, or cerebrovascular disease); (5) pregnancy or breastfeeding; and (6) inability to comply with the study protocol.

### Withdrawal criteria

Withdrawal criteria are as follows: (1) emergence of suicidal tendencies during the study; (2) occurrence of SAEs; and (3) worsening of insomnia that aggravates depressive symptoms.

### Randomization, allocation concealment and blinding

Stratified permuted block randomization will be applied to assign participants to the LA, SLA, and control groups in a 1:1:1 ratio. Stratification will be based on age (18–40 vs. 41–60 years) and gender (male vs. female), resulting in four strata: (1) males aged 18–40 years, (2) males aged 41–60 years, (3) females aged 18–40 years, and (4) females aged 41–60 years. Within each stratum, randomization sequences will be generated using permuted blocks to ensure balance until a total of 40 participants per group are enrolled (120 in total).

The randomization sequence will be generated by an independent research assistant who is not involved in the study, using GraphPad QuickCalcs, a validated web-based program for generating block randomization schemes. Allocation codes will be sealed in sequentially numbered opaque envelopes prepared by independent personnel. At enrolment, envelopes will be opened in sequence by study staff according to the order of patient visits, ensuring allocation concealment. Participants will be informed that they have an equal probability of assignment to each of the three groups.

This study will be conducted as a single-blind trial. Participants, outcome assessors, data collectors, and statisticians will remain blinded to group allocation, while acupuncturists delivering the interventions cannot be blinded due to the nature of the procedures. To maintain blinding integrity, acupuncturists will strictly follow a standardized intervention manual to minimize performance bias.

During treatment, participants will receive therapy in a closed room and will be required to wear blindfolds to prevent observation of laser irradiation. Communication among participants will be restricted, and scheduling will minimize cross-interaction. Researchers will not access unblinded data until data collection and statistical analysis are complete. Allocation concealment and restricted data access will further reduce the risk of bias, thereby strengthening the methodological rigor of the trial.

### Interventions

All participants will continue their stabilized antidepressant regimen throughout the study. Participants in the LA and SLA groups will additionally receive intervention according to a standardized regimen, while those in the control group will receive antidepressant medication only.

**Figure 2:**
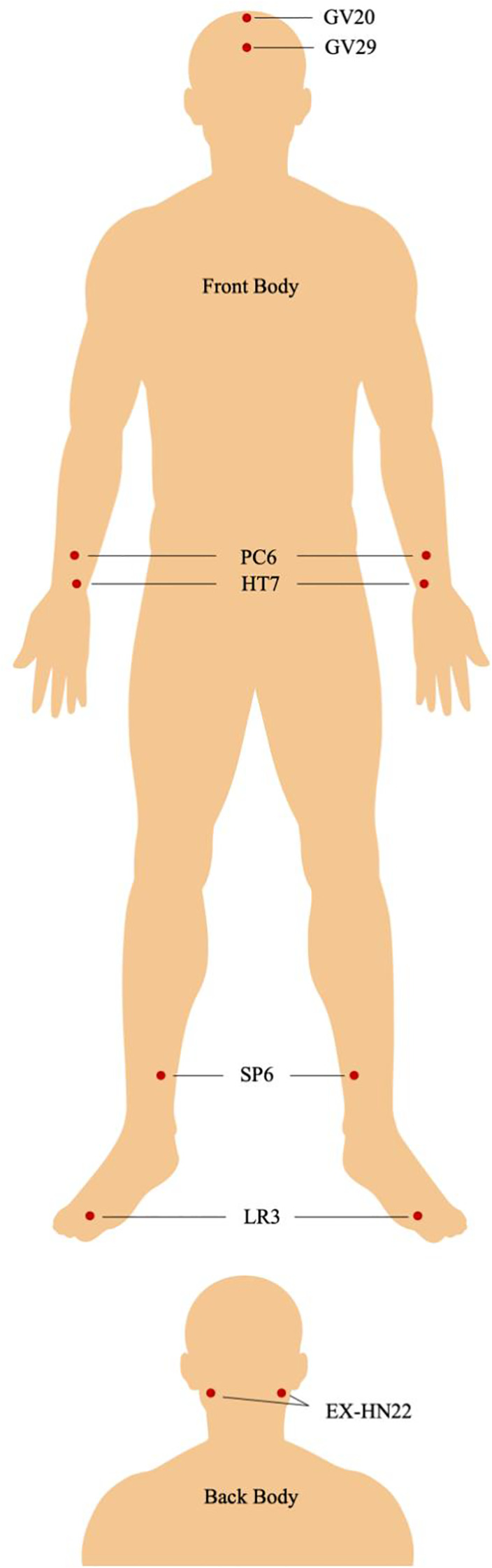
Illustration of the acupoints used in this study. Baihui (GV20): on the vertex of the head, 5 inches posterior to the anterior hairline at the midpoint of the line connecting the apex of both ears; Yintang (GV 29): on the forehead, at the midpoint between the medial ends of the eyebrows; Anmian (EX-HN22): on the posterior aspect of the ear, midway between Fengchi (GB20) and Yifeng (SJ17); Shenmen (HT7): on the wrist, at the transverse crease, in the depression on the radial side of the tendon of flexor carpi ulnaris; Neiguan (PC6): on the forearm, 2 inches above the transverse crease of the wrist between the tendons of palmaris longus and flexor carpi radialis; Sanyinjiao (SP6): on the medial side of the lower leg, 3 inches above the prominence of the medial malleolus, posterior to the medial border of the tibia; Taichong (LR3): on the dorsum of the foot, in the depression distal to the junction of the first and second metatarsal bones.

General procedures (LA and SLA groups). Participants in the intervention arms will receive treatment five days per week (with two rest days) for six weeks, totaling 30 sessions. Each session will last 20 minutes. Before treatment, participants will undergo skin cleansing, wear protective eye masks, and rest in a room maintained at >25°C. Sessions will be delivered by trained acupuncturists at The Second Affiliated Hospital of XXMU who have received protocol-specific instruction to minimize practitioner-related variability. **Figure 2** illustrates the acupoints used in this study and their locations. Paired acupoints will be stimulated bilaterally (Anmian, HT7, PC6, SP6, LR3), whereas midline acupoints will be stimulated at the midline (Baihui/GV20, Yintang/GV29). The acupoints will be used in both the LA and sham LA groups are Baihui (GV20), Yintang (GV29), Anmian (EX-HN22), Shenmen (HT7), Neiguan (PC6), Sanyinjiao (SP6), and Taichong (LR3), selected based on evidence for improving insomnia and depressive symptoms (34–36). Rescue medication is specified under “Medication use across groups” and will be applied uniformly across arms. All use will be documented in the case report form (CRF).

LA procedures. Participants in the LA group will receive low-dose photobiomodulation at prespecified acupoints using the xS-998D06 Semiconductor Laser Acupoint Therapeutic Apparatus (Nanjing Xiaosong Medical Devices Co., Ltd.). The device operates at 650 nm in pulsed mode (50 Hz; 50% duty cycle; stability ≈92%). Dosimetry: reflecting device stability, the on-period output power is 2.3 mW and the time-averaged effective power is 1.15 mW. Each acupoint is irradiated for 20 minutes, delivering 1.38 J per point. The beam spot is standardized with a spacer cap that defines an effective treated area of 1.0 cm², yielding an energy density of 1.38 J/cm². We selected a 1.0 cm² spot to cover the typical footprint of a single acupoint while minimizing spillover, consistent with commonly reported PBM fluence ranges (37). In this trial we apply 12 probes simultaneously, one per acupoint, secured with medical adhesive tape to ensure identical dosing and timing across points and to enhance reproducibility; the total session energy is 16.56 J (1.38 J × 12 acupoints). These low-dose parameters comply with Class 3R laser safety guidance and are supported by prior studies linking photobiomodulation to modulation of 5-HT, sleep quality, and depressive symptoms (38, 39).

SLA procedures. The sham condition used sham laser applied to the same acupoints as the LA group. Identical probes were secured with medical tape and positioned per the standardized manual, but the device delivered 0 mW output while maintaining the same indicators (lights/sounds) to preserve blinding. Session structure (per-point exposure time, total duration, number of sessions, and treatment sequence) matched the active protocol. Participants and outcome assessors were blinded; operators followed a standardized script. Regarding the validity of sham laser as a control procedure for laser acupuncture studies, in a study which evaluated the use of SLA as a control procedure by comparing verum laser with sham laser reported that treatment with verum laser was indistinguishable from sham laser and there was also no difference in the deqi-like sensations felt by study participants when comparing verum laser with sham laser. Then, the authors also analyzed the pooled findings of three RCTs that compared the use of sham laser with needle acupuncture and found that the credibility of sham laser was not different from that of needle acupuncture (39). Hence, sham laser can serve as valid placebo procedure in laser acupuncture studies.

Control group. Participants in the control arm will continue their stable antidepressant regimen without additional intervention. This group establishes a baseline comparator, enhancing the validity of between-group analyses.

Medication uses across groups. Antidepressant therapy must remain stable for ≥2 weeks before enrolment and throughout the trial. Rescue medication will be limited to oral eszopiclone 0.5 mg, to be taken as needed when insomnia persists for ≥3 consecutive nights. No other sedative–hypnotics (including benzodiazepines) will be permitted unless clinically necessary for safety; any exception requires documentation and approval by the principal investigator. All rescue-medication use will be logged (date, time, dose, indication) and reviewed regularly by the research team. If insomnia worsens alongside depressive symptoms, the participant will be withdrawn and referred for further management.

### Treatment fidelity

To ensure consistency, interventions will be administered by licensed acupuncturists with at least three years of clinical experience. In the event of absence, a trained substitute will ensure continuity. Acupuncturists are independent of the research team and blinded to study objectives. Prior to trial initiation, they will complete intensive training workshops on the standardized protocol, including acupoint location, procedural steps, and safety precautions. Training will include demonstrations, role-play, and feedback sessions.

Acupuncturists will be provided with detailed manuals specifying acupoint selection, device application, and treatment timing. Treatment fidelity will be monitored through random review of 15% of recorded sessions by an independent panel of experienced acupuncturists, stratified across providers and intervention stages (early, mid, late). Assessments will follow the revised Standards for Reporting Interventions in Clinical Trials of Acupuncture (STRICTA) guidelines (40), focusing on: (1) accuracy of acupoint location; (2) consistency in timing and session length; (3) adherence to standardized procedures; and (4) inter-rater reliability, measured by Cohen’s Kappa coefficient.

In cases of protocol deviations, the principal investigator will conduct feedback sessions with the involved acupuncturist, provide corrective recommendations, and oversee follow-up monitoring to confirm compliance. Ongoing support will be provided to maintain procedural consistency throughout the trial.

## Outcomes

### Primary outcome

Quality of sleep. The primary outcome is global sleep quality assessed by the PSQI, a 19-item questionnaire widely used to evaluate sleep quality and disturbances over the past month (41). The PSQI includes 4 open-ended and several closed-ended items scored on a 4-point Likert scale, covering subjective sleep quality, sleep latency, sleep duration, sleep efficiency (SE), sleep disturbances, sleep medication use, and daytime dysfunction. The total score ranges from 0 to 21, with higher scores indicating poorer sleep quality and more severe disturbances. A cutoff point of more than 5 is considered as having significant disturbance in sleep quality, which demonstrates a sensitivity of 90% and specificity of 87% (41). The Chinese version of the PSQI has been validated among Chinese medical students, showing acceptable internal consistency (Cronbach’s α = 0.734) (42).

### Secondary outcomes

Insomnia severity. Insomnia severity will be assessed using the Insomnia Severity Index (ISI) (43), a seven-item scale rated from 0 (no problem) to 4 (very severe), with total scores ranging from 0 to 28. Higher scores indicate greater severity: 0–7 = no clinically significant insomnia, 8–14 = subthreshold, 15–21 = moderate, and 22–28 = severe. The Chinese version has shown good reliability in hospital patients (Cronbach’s α = 0.804) (44).

Depression severity. Depressive symptoms will be assessed with the 17-item Hamilton Depression Rating Scale (HAMD-17) (45). Each item is scored on a 3- or 5-point scale, with higher totals indicating greater severity. The Chinese version has demonstrated good reliability (Cronbach’s α = 0.798–0.810) in patients with depression (44). Administration will be performed by an experienced psychiatrist from the Second Affiliated Hospital of XXMU, who is independent of the study team and blinded to study objectives.

Biomarkers. Serum biomarkers will be collected at baseline, post-treatment, and follow-up (08:00 a.m., fasting samples). Levels of 5-HT and CORT, key regulators of sleep and mood, will be quantified using enzyme-linked immunosorbent assay (ELISA) (46, 47).

Actigraphy. Objective sleep parameters will be measured using the ActiGraph wGT3X-BT (ActiGraph LLC, Pensacola, FL, USA), a validated device widely applied in sleep research. Participants will wear the actigraph on the non-dominant wrist for seven consecutive nights, removing it only when necessary to avoid water damage. Data will be collected at 30 Hz and analyzed in 60-second epochs using ActiLife software (version X.X). Sleep–wake scoring will follow the Sadeh algorithm, providing indices such as sleep onset latency, total sleep time, wake after sleep onset, sleep efficiency, nocturnal awakenings, and binary sleep–wake status.

Drug dose record. Participants will complete a daily medication diary from baseline through the 12-week follow-up, documenting drug name, dosage, and exact administration time.

Participant compliance. Adherence to the intervention will be systematically monitored. Reasons for non-compliance (e.g., absences, withdrawal, loss to follow-up, decreased engagement, aversion to treatment, or physical discomfort) will be documented to preserve study rigor.

AEs. AEs will be monitored and classified by severity (mild, moderate, severe). Mild events will be observed; moderate events will trigger timely clinical management; severe events will result in treatment discontinuation and referral to appropriate care. Incidence will be summarized by group, and serious AEs will be followed until resolution.

### Blinding assessment

At the end of the treatment phase, an independent assessor will ask participants in the two intervention groups (LA and SLA) to indicate their perceived allocation by choosing one of three options: LA, SLA, or “I do not know.” Each response will be documented, and the results will be analyzed to evaluate the success of blinding.

The Bang Blinding Index (BBI) will be calculated separately for each intervention group as:


$$BBI_{i}=p_{correct,i}-p_{incorrect,i},i\in \{1,2\}$$

where *p_correct,i_* and *p_incorrect,i _*are the proportions of correct and incorrect guesses in group *i*. “I don’t know” responses are counted in the denominator but not in the numerator. A BBI value between –0.20 and +0.20 will be considered evidence of adequate blinding, whereas values approaching +1 indicate unblinding (48).

### Safety evaluation

Safety will be evaluated throughout the study to ensure participant well-being and to identify potential risks associated with LA, SLA, and control conditions. AEs are defined as any unfavorable or unintended medical occurrence during participation, regardless of its causal relationship to the intervention.

All AEs will be recorded in the CRF, with details on onset, duration, management, and outcome. The investigator will also assess causality, determining whether each AE is related to the intervention. SAEs—such as hospitalization, life-threatening complications, or worsening psychiatric symptoms—will be reported to the ethics committees within 24 hours and followed until resolution or stabilization.

Incidence of AEs will be summarized across groups, and dropout rates due to AEs will be monitored as part of the safety analysis. Participants will receive emergency contact information to report symptoms at any time during the trial. This monitoring framework ensures transparent reporting and timely management of safety concerns.

### Sample size calculation

Sample size estimation was performed using G*Power version 3.1.9.2 (Heinrich Heine University, Düsseldorf, Germany) (49). The calculation was based on a repeated-measures, between–within interaction analysis of variance (ANOVA) with the changes in PSQI across time as the primary outcome. An effect size of f = 0.158 was adopted from a RCT investigating LA in patients with co morbid insomnia, depression and anxiety (50). This effect size was chosen as a conservative estimate, reflecting clinically meaningful but mild improvements in sleep quality. Parameters were set at a two-tailed significance level (α) of 0.05, statistical power (1–β) of 0.80, number of groups = 3 and number of measurements = 3. Under these conditions, the minimum required sample size was calculated to be 84 participants across three arms. To account for an anticipated dropout or attrition rate of approximately 30%, the final adjusted sample size was set at 108 participants, with 36 subjects per group (LA, SLA, and control). This sample size is expected to provide sufficient power to detect between-group differences and to remain robust against missing data or attrition.

### Statistical methods

All analyses will be conducted using statistical package for the social sciences (SPSS) version 28.0 (IBM Corp., Armonk, NY, USA) (51). Continuous variables will be summarized as mean ± standard deviation (SD) for normally distributed data, or as median (interquartile range, IQR) for non-normally distributed data. Categorical variables will be expressed as frequencies and percentages. A two-tailed p value < 0.05 will be considered statistically significant.

Primary outcomes. Changes in PSQI scores, sleep efficiency, total sleep time, and number of awakenings will be analyzed using a mixed-design ANOVA (52), with intervention group (LA, SLA, control) as the between-subject factor and time (baseline, post-treatment, follow-up) as the within-subject factor. Standardized mean differences will be calculated as effect sizes. Significant interactions will be followed by *post-hoc* pairwise comparisons. All primary analyses will follow the intention-to-treat (ITT) principle, with participants analyzed according to initial randomization regardless of adherence or withdrawal.

Secondary outcomes. ISI, HAMD-17, and biomarker levels (CORT, 5-HT) will be analyzed as continuous variables using mixed ANOVA across three time points. Actigraphy-derived sleep parameters will be analyzed similarly. Dropout rates due to AEs will be compared among groups using Pearson’s χ² test or Fisher’s exact test, as appropriate.

A subgroup analysis will be performed in which participants treated with LA will be subdivided into those on selective serotonin reuptake inhibitor (SSRI), serotonin noradrenaline reuptake inhibitor (SNRI) and other antidepressants and on low to moderate dose (less than optimal dose of antidepressant) and on high dose (above the optimal dose of antidepressant) using generalized estimated equation (GEE) where the dependent variable is dummy coded as 0 = without sleep quality disturbance (PSQI ≤ 5) and 1 = with sleep quality disturbance (PSQI > 5).

Sensitivity analysis. A per-protocol analysis will be conducted using the same statistical tests, excluding participants who fail to complete at least 5 of the 6 weekly intervention sessions. Findings will be compared with ITT results.

Missing data. If <5% of data are missing, they will be ignored under the assumption of missing completely at random. When missingness is between 5% and 40%, multiple imputation using chained equations will be applied (Stata 15.0, StataCorp, College Station, TX, USA) (53). If >40% of data are missing or suspected to be non-random, available data will be analyzed with explicit acknowledgment of this limitation.

### Quality control

All investigators will undergo intensive training on the study protocol, quality control procedures, and data management prior to trial initiation to ensure consistency across sites. Only licensed acupuncturists with a minimum of five years of clinical experience will be eligible to deliver the interventions. Before recruitment begins, acupuncturists will receive standardized training in acupoint and non-acupoint localization, intervention procedures, and the operation of sham devices to minimize variability.

A designated clinical supervisor will conduct regular reviews of CRFs, electronic data capture (EDC) records, and adherence to study procedures. In addition, an independent quality control team will be established to perform scheduled and unscheduled monitoring visits. This team will be responsible for verifying data accuracy, ensuring protocol compliance, and addressing any deviations or operational issues in a timely manner. Problems identified during the observation period will be discussed collaboratively, and corrective actions will be implemented to safeguard trial integrity.

### Data collection, management and monitoring

All sociodemographic, clinical, and outcome data will be collected using standardized CRFs and entered an EDC system by trained research staff. Before trial initiation, all personnel involved in data collection and management will undergo uniform training to ensure adherence to standardized procedures for data entry, coding, and validation.

To minimize errors, a double-entry system will be employed in which two independent investigators input the data, followed by cross-checking and discrepancy resolution. Data integrity will be safeguarded through range and logic checks built into the EDC system.

A data monitoring committee composed of statisticians and clinical experts, independent of the study team, will oversee the trial’s conduct and review accumulating safety and efficacy data. Monitoring visits will be performed regularly or on an unscheduled basis to verify source data, assess adherence to the protocol, and address issues promptly.

All trial-related information will be securely stored, with physical documents locked in restricted-access cabinets and electronic data encrypted and password-protected. Access will be limited to authorized investigators only. Data management will be conducted in accordance with the pre-specified data management plan, ensuring transparency, accuracy, and compliance with regulatory and ethical requirements.

### Trial status

The trial was registered on ClinicalTrials.gov (NCT06443242) on June 5, 2024. Recruitment is scheduled to take place from December 2025 to May 2026 at The Second Affiliated Hospital of XXMU, Henan, China. The anticipated trial period will run from December 2025 to June 2026, with follow-up completed by December 2026. Any substantial protocol amendments, including changes in eligibility criteria, intervention details, or outcome measures, will be updated on the trial registry. Results from this study will be submitted for publication in peer-reviewed journals and disseminated at national and international academic conferences. All personal data will be anonymized before dissemination to protect participant confidentiality and prevent individual identification.

## Discussion

This study is designed to investigate the efficacy and safety of LA for insomnia associated with MDD using a RCT design. By assessing sleep quality, depressive symptoms, anxiety, and biomarkers such as serum 5-HT and CORT, this trial aims to generate robust clinical evidence on the role of LA, a minimally invasive intervention, in managing comorbid depression and insomnia (50).

Insomnia and depression are known to have a bidirectional relationship, where sleep disturbance exacerbates depressive symptoms and increases relapse risk (54). Therefore, improving sleep quality is a key therapeutic target in MDD management. LA, as a modern adaptation of traditional acupuncture, is well accepted by patients due to its non-invasive and painless characteristics (55). If shown effective, it may be used alone or in combination with pharmacological treatments, thereby expanding therapeutic options for patients with MDD and insomnia.

We adopted a standardized protocol of seven acupoints, corresponding to 12 total application sites (five paired bilateral points: HT7, PC6, SP6, LR3, EX-HN22; and two midline points: GV20, GV29). The rationale for selecting these points is grounded in both traditional Chinese medicine (TCM) theory and clinical practice (56–58). For instance, HT7 (Shenmen) and SP6 (Sanyinjiao) are widely used for insomnia and emotional disturbances (59), GV20 (Baihui) and GV29 (Yintang) calm the spirit and improve sleep initiation (60), LR3 (Taichong) alleviates tension and regulates mood (61), PC6 (Neiguan) balances the mind and somatic symptoms (62), and EX-HN22 (Anmian) is a classical insomnia point (63). Previous evidence suggests that acupuncture at these points may regulate neurotransmitters and stress hormones (64, 65), supporting improvements in both sleep and mood. Building on this knowledge, our trial extends investigation to the unique photobiomodulation effects of LA, providing novel insights into its therapeutic mechanisms (22, 24, 66).

Serum level of serotonin and cortisol is a window to what happen in the central nervous system (CNS). There is approximately 500 mL of cerebrospinal fluid (CSF) which is absorbed into the bloodstream from the CNS daily. Presence of psychiatric illness such as depression may lead to blood brain barrier dysfunction which allow increasing exchange of proteins between peripheral blood and CSF, which include hormone like cortisol and neurotransmitter such as serotonin. Hence, the serum cortisol and serotonin may represent what actually occurred in the brain (67).

This study is expected to contribute theoretically by integrating clinical outcomes with biomarker analyses to elucidate how LA may modulate both peripheral and central pathways involved in sleep and mood regulation (64, 67). Such findings may help establish a framework for incorporating non-invasive acupuncture techniques into holistic mental health care.

Several limitations should be acknowledged. First, recruitment from a single center may restrict the generalizability of results. Moreover, the effect size for estimation of sample size in this study was conservatively estimated and the sample size assumptions with reference to a previous RCT on effectiveness of LA for treatment of co morbid depression, anxiety and insomnia (50). Hence, this may also affects the generalizability of the study findings. Second, the relatively short follow-up period limits evaluation of long-term efficacy. Third, despite efforts at standardization, variability in acupuncturist training and resource allocation may introduce bias. Fourth, while standardized acupoint selection ensures reproducibility, it does not reflect individualized syndrome differentiation in TCM, which may be explored in future studies (68, 69). Fifth, although the sham laser procedure is operationally indistinguishable from the LA procedure, a pilot study for blinding assessment of the sham laser procedure is not performed. Hence, the risk of bias and compromise of blinding remains unresolved. Finally, the application of sham laser at identical acupoints as that of LA may induce non-specific physiological effects unrelated to laser output as the sham laser stimulate the acupoint area through pressure or contact which leads to activation of mechanoreceptors or nerve fibers that trigger neurological modulation, changes in blood flow or endorphin release (70, 71).

Future research should therefore consider multi-center trials, longer follow-up, and cross-cultural collaboration to enhance external validity. Studies may also examine the long-term effects of LA, optimize treatment parameters (e.g., wavelength, dose, frequency), and assess its synergistic potential with pharmacological or behavioral interventions. In addition, advanced neuroimaging and biomarker analyses could further clarify its neurobiological mechanisms.

This trial addresses an important therapeutic gap by evaluating LA, a novel and non-invasive intervention for insomnia comorbid with MDD. By integrating both clinical outcomes and biomarker analyses, the study is expected to contribute to a deeper understanding of how LA may benefit patients with sleep and mood disturbances. These findings could broaden treatment strategies and promote the adoption of safe, non-pharmacological options within comprehensive mental health care.

## Conclusion

In conclusion, this study has been designed with rigorous methodology, including strict adherence to the CONSORT statement and revised STRICTA guidelines, to ensure transparency and reproducibility. By systematically assessing sleep quality, depressive symptoms, and biological markers, the trial will provide high-quality evidence on the efficacy, safety, and feasibility of LA. The findings are anticipated to inform clinical practice and guide the development of accessible, evidence-based treatment strategies for patients with MDD and insomnia.

## Glossary

5-HT: 5-Hydroxytryptamine (Serotonin)
AE: Adverse Event
ANOVA: Analysis of Variance
BBI: Bang Blinding Index
CBT-I: Cognitive Behavioral Therapy for Insomnia
CONSORT: Consolidated Standards of Reporting Trials
CORT: Cortisol
CRF: Case Report Form
DSM-5-TR: Diagnostic and Statistical Manual of Mental Disorders, Fifth Edition, Text Revision
EDC: Electronic Data Capture
ELISA: Enzyme-Linked Immunosorbent Assay
HAMD-17: 17-item Hamilton Depression Rating Scale
HPA: Hypothalamic–Pituitary–Adrenal
IQR: Interquartile Range
ISI: Insomnia Severity Index
ITT: Intention-to-Treat
LA: Laser Acupuncture
MDD: Major Depressive Disorder
PTSD: Post-Traumatic Stress Disorder
PSQI: Pittsburgh Sleep Quality Index
RCT: Randomized Controlled Trial
SAE: Serious Adverse Event
SD: Standard Deviation
SLA: Sham Laser Acupuncture
SPIRIT: Standard Protocol Items: Recommendations for Interventional Trials
SPSS: Statistical Package for the Social Sciences
STRICTA: Standards for Reporting Interventions in Clinical Trials of Acupuncture
TCM: Traditional Chinese Medicine
USM: Universiti Sains Malaysia
XXMU: Xinxiang Medical University.
