# Supplementary material for: Efficacy and safety of laser acupuncture for treating insomnia in major depressive disorder: study protocol for a randomized controlled trial
Source: Front Psychiatry. 2025 Nov 20;16:1698773. doi: 10.3389/fpsyt.2025.1698773 (PMC12676490; doi:10.3389/fpsyt.2025.1698773)
Supplement: Supplementary file 2 [file DataSheet2.pdf]

Appendix B: CONSORT 2025 Flow Diagram

Flow diagram of the progress through the phases of a randomised trial of two groups (that is, enrolment, intervention allocation, follow-up, and data analysis)

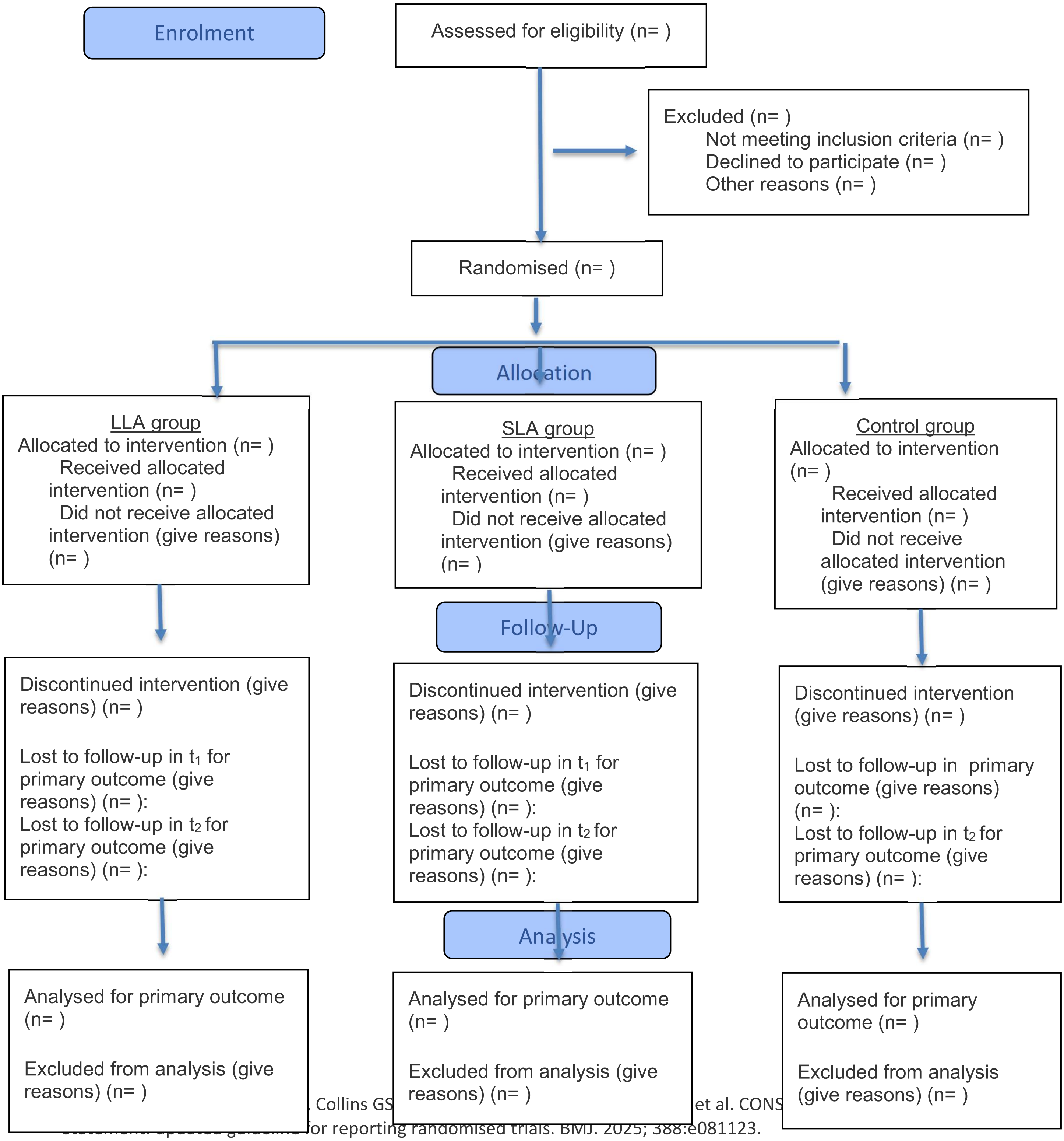

[illegible] Collins GS [illegible] et al. CONS[illegible] [illegible] for reporting randomised trials. BMJ. 2025; 388:e081123.
https://dx.doi.org/10.1136/bmj-2024-081123

© 2025 Hopewell et al. This is an Open Access article distributed under the terms of the Creative Commons Attribution License (https://creativecommons.org/licenses/by/4.0/), which permits unrestricted use, distribution, and reproduction in any medium, provided the original work is properly cited.
