# Supplementary material for: Efficacy and safety of laser acupuncture for treating insomnia in major depressive disorder: study protocol for a randomized controlled trial
Source: Front Psychiatry. 2025 Nov 20;16:1698773. doi: 10.3389/fpsyt.2025.1698773 (PMC12676490; doi:10.3389/fpsyt.2025.1698773)
Supplement: Supplementary file 3 [file DataSheet3.pdf]

Supplementary appendix 3. Research protocol approved by the Institutional Review Board

Research title: Efficacy and Safety of Low-dose Laser Acupuncture on Treating Insomnia Associated with Major Depressive Disorder: A Randomised Controlled Trial

Principal investigator (MMC No. if applicable): Qiu Qiyue, Dr. Mohammad Farris Iman Leong Bin Abdullah, Assoc Prof Dr. Yam Mun Fai, Prof. Yang Shichang

Co-researchers: (MMC No. if applicable): -

Introduction

1.1 ***Background of the study***

Depression and insomnia are highly prevalent and interrelated mental health disorders that profoundly impact individual well-being and overall quality of life. Depression characterised by persistent sadness, loss of interest, and impaired functioning, affects over 264 million people globally, making it one of the leading causes of disability worldwide (James et al., 2018; Kessler et al., 2005; WHO, 2017). In China alone, over 50 million individuals, approximately 3.6% of the population, experience depressive disorders annually, underscoring its significance in public health domains (Huang et al., 2019; Ferrari et al., 2013).

Insomnia, manifesting as difficulty initiating or maintaining sleep, frequent awakenings during the night, or experiencing non-restorative sleep, frequently coexists with depression. Studies have shown that about 70% of individuals with depression suffer from insomnia symptoms, and the prevalence of insomnia in those with depression is three to four times higher than in those without depression (Sunderajan et al., 2010; Andrade et al., 2003; Baglioni et al., 2011). This bidirectional relationship exacerbates the challenges faced by affected individuals, affecting their ability to function effectively in daily life.

The interplay between depression and insomnia not only challenges individual functioning but also poses significant socio-economic impacts. For instance, the

lifetime prevalence of major depressive disorder in China has been reported at 16%, highlighting the urgent need to enhance healthcare services for affected individuals (Andrade et al., 2003; Kessler et al., 2003).

1.1.1 *Pathogenesis and clinical features of insomnia associated with depression*

The interplay between depression and insomnia involves complex pathophysiological mechanisms and clinical features that deeply intertwine these conditions. Epidemiologically, common risk factors for both insomnia and depression include ageing, gender differences with females being more susceptible, and socio-economic factors such as divorce, widowhood, and unemployment. Additionally, life stressors and a lack of social support also contribute significantly (Sivertsen et al., 2012; Li et al., 2020).

Neuroendocrine changes: In patients with depression, there is heightened activity of the hypothalamic-pituitary-adrenal (HPA) axis, which leads to abnormal cortisol secretion. This dysregulation affects sleep patterns, mainly changes in rapid eye movement (REM) sleep (Pace-Schott et al., 2012). The dysfunction of the HPA axis is also considered a critical biomarker for the comorbidity of depression and insomnia (Gold et al., 2002).

Alterations in sleep architecture: Those suffering from depression frequently experience disruptions in sleep continuity, such as extended sleep latency and frequent awakenings throughout the night. There is a reduction in slow-wave (deep) sleep, with notable changes in REM sleep, including an earlier onset and increased REM density (Palagini et al., 2013).

Changes in neurotransmitters: research has shown that neurotransmitters like serotonin (5-HT) play a crucial role in the modulation of both depression and insomnia. Alterations in the 5-HT1A and 5-HT2A receptors are closely associated with regulating sleep architecture and depressive states (Jansson-Fröjmark & Lindblom, 2008).

Neuroimaging findings: PET scans have demonstrated that patients with depression and insomnia exhibit reduced thalamocortical activity during non-REM sleep while showing increased activity in specific brain regions during REM sleep. These findings reflect different regulatory mechanisms of brain function under these conditions (Nofzinger et al., 2004).

The robust and bidirectional relationship between depression and insomnia complicates clinical management, as insomnia not only exacerbates depression symptoms but also serves as a predictor for the development of depressive episodes. Persistent insomnia is particularly harmful, significantly raising the likelihood of chronic depression, which is more challenging to treat and carries a higher risk of relapse (Coryell et al., 2013; Kato, 2014). Additionally, insomnia heightens the risk of suicidal ideation, further emphasising the need for effective therapeutic strategies that address both insomnia and depression to mitigate these severe outcomes (Suh et al., 2013; Lapierre et al., 2012).

In treating these intertwined conditions, improving sleep quality through various interventions often leads to a notable reduction in depressive symptoms, suggesting that addressing insomnia may also alleviate the depressive state itself (Sivertsen et al., 2012; Nadorff et al., 2013). This cyclical interplay highlights the complexity of diagnosis and treatment, demanding a multifaceted approach to manage both conditions simultaneously.

1.1.2 Clinical treatment of insomnia associated with depression and their limitations

a. Western Medical Treatments:

- Pharmacotherapy: Typically, antidepressants and hypnotics are prescribed. While effective for some, these medications often lead to dependency and adverse effects such as cognitive impairment, undermining long-term usage (Lader et al., 2009; Sasai et al., 2010).

- Cognitive Behavioral Therapy for Insomnia (CBT-I): This non-pharmacological approach is effective but underutilised, often due to cultural barriers and resource limitations (Ng & Wong, 2018).

b. Traditional Chinese Medicine (TCM):

- Acupuncture: This ancient technique has shown promise in treating depression-related insomnia by modulating neurotransmitters and inflammatory mediators (Dong et al., 2017; Zhao et al., 2021). Despite its therapeutic benefits, acupuncture may involve some limitations, such as pain, bleeding, needle breakage, and patient anxiety towards the treatment (MacPherson et al., 2001; White, 2004).

- Laser Acupuncture: As a modern adaptation, laser acupuncture offers a non-invasive treatment alternative, stimulating acupoints through irradiation with limited pain. It is particularly suitable for those with needle phobias or sensitivity, including children and the elderly (Litscher, 2018; Jang et al., 2019).

Challenges and Future Directions:

Despite these treatments, the management of comorbid depression and insomnia remains challenging due to the complexity of their interaction. Pharmacotherapy, although commonly employed, faces significant drawbacks in terms of side effects and dependency risks. On the other hand, non-drug therapies like CBT-I and acupuncture require more widespread acceptance and accessibility to become standard care components.

Furthermore, while acupuncture (both traditional and laser) shows promise, conclusive evidence through rigorous randomised controlled trials is still needed to firmly establish its efficacy and safety for depression-associated insomnia (Li et al., 2022). The limitations associated with traditional acupuncture, such as discomfort and fear of needles, highlight the need for further innovation and patient education in this area.

Problem statement & Study rationale

Patients suffering from depression often experience clinically significant insomnia, which tends to exacerbate the severity of their depressive symptoms and complicates treatment efforts. Current mainstays of treatment—pharmacotherapy and psychotherapy—present significant challenges, including a scarcity of therapists, considerable financial burdens for patients, and a plethora of side effects from medications, such as dependency, tolerance, adverse reactions, and withdrawal symptoms upon discontinuation. Notably, certain antidepressants may exacerbate insomnia or induce excessive daytime sleepiness, further complicating treatment outcomes. Consequently, the limitations of existing treatments for depressive disorders-related insomnia have driven an increasing number of patients towards complementary and alternative medicine (CAM), seeking practical and safer alternatives.

Given the limitations and adverse effects associated with conventional treatments for insomnia associated with depression, there is a compelling need for alternative therapeutic options. Laser acupuncture, a non-invasive form of traditional acupuncture, emerges as a promising candidate due to its potential for alleviating symptoms without the discomfort or risks associated with needle insertion, such as pain, bleeding, or broken needles. The foundation of laser acupuncture in Low-Level Laser Therapy (LLLT) offers a bio-stimulation method that mirrors traditional acupuncture's benefits but eliminates the invasiveness and associated complications. Despite the growing body of research indicating acupuncture's efficacy and safety for treating insomnia and its capacity to improve depressive symptoms, the evidence remains inconclusive, particularly regarding its effect on insomnia associated with depressive disorders. This ambiguity highlights the necessity for rigorous randomised controlled trials (RCTs) to ascertain laser acupuncture's effectiveness and safety in this context. Such research is crucial not only for validating laser acupuncture as a treatment modality but also for exploring its underlying mechanisms, potentially

involving the regulation of critical neurotransmitters and stress hormones like serotonin (5-HT) and corticosterone (CORT), which play significant roles in the pathophysiology of both insomnia and depressive disorders.

Research Question(s)

1. How effective is LLA in alleviating insomnia symptoms as compare with SLA and control subjects among patients with major depressive disorder across pre-treatment, mid-treatment and post-treatment assessment?

2. What role do CORT and 5-HT play in the co-occurrence and progression of insomnia and major depressive disorder, and how does LLA act on these mechanisms to provide relief?

3. Does LLA enhance the effectiveness of pharmacological interventions in treating insomnia and major depressive disorder when used as an adjunctive treatment?

4. How does the safety and acceptability of LLA compare to traditional acupuncture in terms of eliminating discomfort and potential side effects?

Objective

Primary objective:

To assess the efficacy and safety of low-dose laser acupuncture (LLA) in mitigating insomnia symptoms (sleep efficiency, frequency of awakenings during sleep, and total duration of sleep, coupled with an examination of the severity of insomnia) as compare with SLA and control subjects among patients with major depressive disorder across pre-treatment, immeditely post-treatment and 12 weeks post-treatment assessment.

Secondary objectives:

1. To evaluate the impact of LLA on a range of measures, such as the severity of depressive and anxiety symptoms as compare with SLA and control subjects among

patients with major depressive disorder across pre-treatment, immediately post-treatment and 12 weeks post-treatment assessment.

2. To investigate the variations in serum levels of Corticosterone (CORT) and 5-hydroxytryptamine (5-HT) in LLA subjects as compare with SLA and control subjects among patients with major depressive disorder across pre-treatment, immediately post-treatment, and 12 weeks post-treatment assessment.

3. To compare the insomnia outcome derived from varying treatment amalgamations, which include LLA and antidepressant drug-aided therapy (LLA + SSRI vs. LLA + mirtazapine vs. LLA + SNRI).

4. To investigate the safety of laser acupuncture on patients immediately post-treatment and 12 weeks post-treatment (comparison of dropout rate due to adverse effects between LLA, SLA and control groups).

Hypotheses:

H_1: The efficiency and duration of sleep significantly increase and frequency of awakening and severity of insomnia significantly decrease in the LLA group compared with SLA and control groups from pre-treatment to immediately post-treatment and 12 weeks post-treatment assessment.

H_2: The severity of depression and anxiety symptoms significantly decrease in the LLA compared with the SLA and control group from pre-treatment to immediately post-treatment and 12 weeks post-treatment assessment.

H_3: The serum level of cortisol and serotonin significantly decrease in the LLA group compared with the SLA and control groups from pre-treatment to immediately post-treatment and 12 weeks post-treatment assessment.

H_4: The efficiency and duration of sleep documented greatest increase and frequency of awakening and severity of insomnia documented greatest decrease in

the LLA + mirtazapine subgroup compared with LLA + SSRI and LLA + SNRI groups at immediately post-treatment and 12 weeks assessment.

H_5: The dropout rate due to adverse effects of the LLA group is comparable to the SLA and control groups at immediately post-treatment and 12 weeks post-treatment assessment

Literature review

2.1 Depressive Disorders and Sleep Disturbances

Depressive disorders, characterised by symptoms such as depressed mood, loss of interest, and reduced energy resulting in diminished activity, represent one of the most prevalent psychiatric diagnoses, impacting over 264 million individuals globally (James et al., 2018). More than 50 million individuals in China, constituting approximately 3.6% of the population, experience depressive disorders each year (Huang et al., 2019).

Depression ranks among the prevalent psychiatric conditions in adults and is highlighted as the second-highest contributor to global disability (Ferrari et al., 2013). In China, the estimated lifetime prevalence of major depressive disorder (MDD) is reported at 3.3%, emphasising the pressing necessity to enhance healthcare services for affected individuals. Depressive symptoms encompass negative emotions, anxiety, agitation, low self-esteem, and suicidal tendencies (Van Wingen et al., 2011).

MDD often co-occurs with sleep disturbances like insomnia or hypersomnia (Murphy & Peterson, 2015). Insomnia, prevalent in 80–90% of individuals with MDD, presents as persistent dissatisfaction with sleep quantity or quality (Srisurapanont et al., 2015). Even after mood symptoms have been adequately treated, insomnia often persists for many patients, leading to significant challenges in daily functioning (Yin et al., 2016).

Depression also constitutes a significant portion of the disease burden in China, second only to another significant ailment in terms of financial impact (Greenberg & Birnbaum, 2005; Ferrari et al., 2013). Recent epidemiological surveys revealed a lifetime occurrence rate of depression at 16% (Andrade et al., 2003; Kessler et al., 2003).

Insomnia, a prominent symptom among individuals with depression, is characterised by challenges in both falling asleep initially and maintaining sleep throughout the night (Breslau et al., 1996; Saarni et al., 2007). It leads to reduced sleep duration and diminished sleep quality, impacting one's capacity to learn and work effectively and overall quality of life. The connection between depression and sleep disorders is intricate and multifaceted (Breslau et al., 1996).

Insomnia and depression frequently intertwine, with around 70% of individuals with depression experiencing symptoms of insomnia (Sunderajan et al., 2010). The prevalence of depression among individuals with insomnia surpasses that of those without insomnia by 3 to 4 times (Andrade et al., 2003). Moreover, sleep disorders serve as prominent indicators and diagnostic criteria for depression (Dudek & Koniarek, 2000). A symptom of severe depression can include waking up at least two hours earlier than usual (Andrade et al., 2003).

2.2 Symptoms, Implications, and Treatment of Insomnia

Sleep is crucial for the body's recuperation from exhaustion and repair processes (Fox, 1999; Lee et al., 2009). Sufficient sleep aids in restructuring both the body and mind after daily tasks (Dement, 2010). However, chronic sleep insufficiency, or diminished sleep quality, is linked to numerous physical and psychological conditions, encompassing weakened immunity, hormonal disorders, emotional fluctuations, attention deficits, decreased job efficiency, and an elevated likelihood of mishaps (Orzeł-Gryglewska, 2010). Sleep disruptions hold significant implications for community well-being.

Typically, sleep ailments are addressed with drug treatments. Nevertheless, these can lead to unwanted, even perilous, consequences like short-term memory lapses, lethargy during the day, imbalance, and walking challenges (Tariq & Pulisetty, 2008; Yang et al., 2011). Insomnia is a prevalent sleep disorder characterised by unsatisfactory sleep, difficulty falling or staying asleep, and premature awakenings. This condition can hamper daily activities, leading to exhaustion, melancholy, and stress. While drugs are frequently used to treat insomnia, they might result in unwanted outcomes such as mental and physical addiction, lingering drowsiness during the day, and cognitive disturbances (Wang et al., 2022).

Insomnia is the most common sleep disturbance in medical practice (Schutte-Rodin et al., 2008). It encompasses challenges in initiating and maintaining sleep, early awakening, frequent arousals, and difficulties returning to sleep, all accompanied by impaired daytime functioning. This may manifest as sleepiness, irritability, tiredness, fatigue, and cognitive impairments like poor concentration and memory (Roth, 2007; Punnoose et al., 2012; Merrigan et al., 2013; Taylor et al., 2014). The prevalence of insomnia ranges from approximately 6% to 30% in the general population (Ohayon, 1997; Roth & Ancoli-Israel, 1999; Roth, 2007).

Insomnia has been linked to heightened morbidity for both somatic and mental conditions, particularly depressive disorder. It correlates with elevated healthcare utilisation, higher rates of absenteeism from work, reduced productivity, and an increased risk of industrial and vehicular accidents (Roth, 2007; Bogle et al., 2009; Daley et al., 2009). Indeed, evidence indicates that insomnia serves as an independent risk factor for suicide (Lin et al., 2018).

Patients grappling with insomnia frequently endure daytime repercussions such as sleepiness, fatigue, diminished concentration, mood alterations, and disruptions in family dynamics and social interactions (Medina-Chávez et al., 2014). According to the third edition of the International Classification of Sleep Disorders (ICSD), "Insomnia disorder is characterised by difficulty initiating or maintaining sleep,

accompanied by daytime consequences. Importantly, these symptoms are not solely attributable to environmental circumstances or inadequate opportunities to sleep." (Sateia, 2014).

Epidemiological studies suggest that the prevalence of chronic insomnia in developed nations ranges from 5% to 10% (Ohayon, 2009). Chronic insomnia is linked to significant impairments in both function and quality of life (Spira et al., 2014). Insomnia is characterised by diminished sleep quality or quantity, challenges initiating sleep, and frequent awakenings at night or early morning.

It is widely acknowledged that there exists a strong association between depression and insomnia. Specifically, patients with depression often experience abnormal alterations in their typical sleep-wake cycle, with sleep disturbances frequently being the primary complaint (Armitage, 2007). Considering that depression can markedly disrupt the usual sleep patterns, persistent insomnia emerges as one of the most prominent symptoms in individuals with depressive symptoms (Ohayon & Roth, 2003). Reports indicate that approximately 60–80% of individuals with depression experience persistent insomnia (Winokur et al., 2001). Even after depressive symptoms have been adequately alleviated, reduced sleep quality and quantity may persist, exacerbating the overall condition.

Treatment for sleep disorders typically involves pharmacotherapy, but this approach can lead to undesirable and potentially hazardous side effects, including temporary memory impairment, daytime drowsiness, impaired balance, and difficulties with gait (Tariq & Pulisetty, 2008; Yang et al., 2011). As a result, individuals with sleep disorders often harbour negative sentiments toward the use of hypnotic medications and are open to exploring non-pharmacological therapies.

2.3 The Interplay Between Depression and Insomnia

Numerous studies have identified a robust connection between depression and insomnia, with insomnia serving as a significant predictor of changes in depression

over time (Cheung & Wong, 2011; Wright et al., 2011; Okajima et al., 2012; Sivertsen et al., 2012; Coryell et al., 2013; Kato, 2014). Patients experiencing persistent insomnia often exhibit significantly elevated rates of depression and suicidal ideation (Suh et al., 2013). Furthermore, non-refreshing sleep and difficulties in both initiating and maintaining sleep are correlated with an elevated risk of depression (Salo et al., 2012). The association between suicidal ideation and insomnia might be influenced by depressive symptoms (Lapierre et al., 2012; Nadorff et al., 2013).

There is a growing acceptance of the bidirectional relationship between depression and insomnia (Fang et al., 2019). Despite conventional treatment approaches, managing depression-related insomnia remains a challenge. Emerging evidence suggests that acupuncture may offer a promising avenue for addressing this complex condition (Dong et al., 2017; Zhao et al., 2021). While the precise mechanisms behind acupuncture's efficacy remain incompletely understood, it is believed to operate through intricate pathways, including modulation of neurotransmitters and inflammatory mediators (Lu et al., 2017; Zhao et al., 2021).

In recent years, further exploration of the bidirectional relationship between depression and insomnia has occurred. Insomnia can heighten the risk of depression, subsequently worsening insomnia. Persistent insomnia is now recognised as an , k,independent diagnostic entity capable of precipitating and exacerbating depressive disorders. Addressing insomnia has been shown to enhance the clinical outcomes of depression (McCall et al., 2010). Thus, there is a growing focus on addressing the sleep-specific treatment requirements of patients with depression.

Depression and insomnia share a reciprocal link, posing a significant medical challenge. While acupuncture emerges as a potential therapeutic avenue, further research, particularly randomised controlled trials (RCTs), is needed to establish its impact and safety for depressive disorders-linked insomnia (Li et al., 2022). Consequently, there is a need for well-designed RCTs, such as placebo-controlled studies, to explore the impact of interventions like laser acupuncture on insomnia,

with a focus on enhancing sleep quality and alleviating psychological symptoms, predominantly depressive disorders.

Depression and sleep issues share a bidirectional relationship. Poor sleep quality can contribute to the onset of depression, and individuals with depression are more prone to developing sleep issues (Matsuda et al., 2017; Difrancesco et al., 2019). Comorbid depression and sleep disorders pose challenges in treatment. They are associated with an increased risk of suicide, severe impairment in social functioning, and higher rates of relapse and recurrence of depression (Nutt et al., 2008; Fang et al., 2019; Wang et al., 2019; J. Zhao et al., 2021).

2.4 Treatment Options for Insomnia

In Western medicine, insomnia is commonly managed through non-pharmacological strategies, cognitive behavioural therapy (CBT), and medication. Non-pharmacological approaches and CBT are favoured for chronic insomnia while sleeping pills are reserved for acute cases. However, prolonged medication use can lead to addiction and adverse effects, deteriorating quality of life (Sasai et al., 2010). Insomnia symptoms are associated with increased psychotropic medication use, further highlighting the need for effective alternatives (Haaramo et al., 2014). Acupuncture has emerged as a promising option, demonstrating efficacy without adverse effects (Ling et al., 2008; Jiang et al., 2010; Zhou et al., 2012).

Pharmacotherapy, though typical for chronic insomnia, is fraught with side effects, including dependency and cognitive impairment (Lader et al., 2009). Many individuals seek complementary and alternative medicine (CAM) therapies like acupuncture for insomnia relief, reflecting a growing trend (Bertisch et al., 2012). However, the management of comorbid depression and insomnia often relies on pharmacotherapy despite its limitations (Cardinali et al., 2012; Chan et al., 2015; Chien et al., 2015). Acupuncture offers a non-pharmacological alternative with promising results in improving sleep quality and duration (Cheuk et al., 2007).

Traditional acupuncture and its non-invasive counterpart, laser acupuncture, have shown efficacy in managing insomnia (Maciocia, 2013). Acupuncture's mechanism regulates neuroendocrinological factors, promoting physiological balance (Zhao, 2013). However, past studies on acupuncture's efficacy in treating depression alongside insomnia yield mixed results, necessitating further research (Chung et al., 2018).

Cognitive behavioural therapy for insomnia (CBT-I) is effective but underutilised due to cultural barriers and limited resources (Ng & Wong, 2018). Antidepressants, commonly prescribed for depression-related insomnia, pose challenges, including dependency and sedation (Gibson-Smith et al., 2015; Wichniak et al., 2017). Acupuncture, with its minimal side effects and long history, provides an attractive alternative (Dong et al., 2017; Zhao et al., 2021; Liu et al., 2021).

Acupuncture's effectiveness in depression treatment, combined with its role in managing insomnia, makes it a valuable therapeutic tool (Chan et al., 2015). However, conclusive evidence of its efficacy for depression-related insomnia is lacking, necessitating further research (Yin et al., 2019). Despite challenges, acupuncture holds promise as a safe and effective treatment option for insomnia, especially when comorbid with depression.

2.5 Understanding Acupuncture for Insomnia and Depression

Acupuncture, originating from Traditional Chinese Medicine (TCM), has a rich history spanning over 2500 years, with its fundamental principle revolving around the concept of Qi, or inner energy, and restoring balance in its circulation (Chon & Lee, 2013). In contemporary practice, traditional acupuncture involves the insertion of thin needles into specific acupoints on the skin, while electroacupuncture utilises electric currents between these needles (Chon & Lee, 2013).

The bidirectional relationship between insomnia and negative emotions underscores the importance of addressing both conditions simultaneously. Acupuncture has

shown promise in managing insomnia and its associated psychological symptoms by targeting specific acupoints, including Anmian points behind the ears (Huo et al., 2013). By combining acupuncture at meridian acupoints with stimulation of Anmian acupoints, improvements in sleep quality, depression, and anxiety have been observed (Huo et al., 2013).

Acupuncture therapy may aid in inducing sleep by alleviating anxiety, making it a favourable option over Western medication, often with fewer adverse reactions (Luo et al., 2010; Lu & Lu, 2013). It regulates melatonin secretion, improving insomnia symptoms and reducing anxiety scores (Spence et al., 2004). Acupuncture's efficacy in treating negative emotions associated with insomnia may be attributed to its ability to enhance brain wave activity and regulate the flow of Qi (Zhou et al., 2013).

In traditional Chinese medicine theory, depression is often viewed as a "yin" disease, and its treatment involves regulating the "yang" meridians, such as the Governor Vessel (Zhou et al., 2013). Acupoints like Shenmen (HT7), Baihui (DU20), Hegu (LI4), and Taichong (LR3) have been identified for their effectiveness in calming the mind and improving symptoms of insomnia and depression (Luo et al., 2010). Acupuncture treatment significantly enhances brain wave activity, further contributing to its therapeutic effects (Zhou et al., 2013).

Studies have identified specific acupoints, such as Shenmai (BL62) and Zhaohai (KI6), along with others like Baihui (DU20), Sishencong (EX6), and Zusanli (ST36), commonly used in treating insomnia (Lin et al., 2016). Acupuncture prescriptions aimed at regulating the yin-yang balance and the functions of the five viscera have shown effectiveness in addressing insomnia (Lin et al., 2016).

Despite the growing research on acupuncture's efficacy for depression and insomnia, there is a need for more studies, especially investigating the effects of Laser Acupuncture (LA) on concurrent depression and insomnia (Yang & Zhai, 2013). Additionally, integrating acupuncture theory with practitioners' expertise can

optimise treatment approaches, prioritising methods like electroacupuncture targeting specific acupoints for enhanced therapeutic outcomes (Yin et al., 2016).

In summary, acupuncture offers a holistic and complementary approach to managing insomnia and its associated complications, rooted in centuries-old wisdom and increasingly supported by scientific evidence.

2.6 Exploring Laser Acupuncture for Depression-Associated Insomnia

Laser acupuncture, a contemporary advancement in acupuncture practice, offers a non-invasive and gentle alternative to traditional needle therapy. It involves the stimulation of acupoints through irradiation, triggering physiological effects at the cellular level (Litscher, 2018). Originating from China and Russia, laser acupuncture gained clinical application in the 1970s. It has since gained traction due to its user-friendly nature, particularly among individuals with needle phobias, the elderly, and children (Jang et al., 2019).

The ideal parameters for laser acupuncture, as outlined by Moskvin and Agasarov (2020), include specific wavelengths, power outputs, and exposure times, ensuring effective stimulation of acupoints while minimising risks. Following prescribed protocols, the methodical administration of laser acupuncture ensures safe and consistent treatment delivery.

Depression, a prevalent global health concern, presents significant challenges in both diagnosis and treatment. Despite efforts to raise awareness, many individuals with depression opt to manage symptoms independently, often turning to complementary and alternative medicines (CAMs) due to concerns about adverse effects associated with conventional antidepressants (Ellis & Smith, 2002; MacPherson et al., 2006).

Antidepressant medications, while commonly prescribed, have limited efficacy, with a considerable proportion of patients discontinuing treatment due to adverse effects

(Pigott et al., 2010). This has spurred interest in exploring non-drug treatments for depression, including acupuncture. However, the scientific evidence base regarding the efficacy of acupuncture for depression remains inconclusive, with methodological issues complicating many studies (Mukaino et al., 2005; Leo & Ligot, 2007; Halbreich, 2008; Smith et al., 2018).

Laser acupuncture, with its non-invasive and user-friendly nature, presents a promising avenue for depression treatment. A pilot study by Quah-Smith et al. demonstrated the effectiveness of laser acupuncture compared to placebo laser for treating mild to moderate depression, with sustained treatment response observed over three months (Quah-Smith et al., 2005). Functional magnetic resonance imaging (fMRI) studies further support the efficacy of laser acupuncture, showing significant activation and deactivation in relevant brain regions associated with mood regulation (Quah-Smith et al., 2010).

In conclusion, laser acupuncture offers a potentially valuable adjunctive therapy for depression, providing a safe and effective alternative to conventional treatments. Further research is needed to elucidate its mechanisms of action and optimise treatment protocols for enhanced clinical outcomes.

2.7 Application of Low-dose Laser Therapy

In recent years, laser acupuncture (LA) or low-dose laser therapy (LLT) has emerged as a promising alternative to traditional needle acupuncture, offering a non-invasive and pain-free method of stimulating acupoints. Originating in the 1970s with successful treatments for conditions like asthma and hypertension in the Soviet Union, LA has gained popularity due to its potential benefits, especially for specific patient populations such as geriatric and paediatric patients (Gamaleya, 1977).

Compared to traditional metal needles, LA sessions typically have shorter durations, ranging from 10 to 60 seconds per acupuncture site, significantly reducing overall

treatment time (Hu et al., 2014). This makes LA particularly appealing for individuals with limited tolerance for longer acupuncture sessions.

Safety is another advantage of LA, especially for accessing challenging acupoints in regions like the thoracic cage, where traditional needle acupuncture may pose a higher risk of complications such as pneumothorax (Chen et al., 2015). Research indicates that LA is as effective as conventional acupuncture in addressing various conditions, including myofascial pain, postoperative nausea/vomiting, and chronic tension headaches (Zhao, 2013).

In LA sessions, acupoint selection follows the fundamental principles of Chinese medicine, particularly the concept of Yin and Yang. The balance between Yin and Yang is crucial for maintaining health, with disruptions leading to various health issues, including insomnia. Insomnia, often characterised by an imbalance between Yin and Yang, can stem from deficiencies or disharmony in various organs and meridians, disrupting the body's equilibrium and causing sleep disturbances (Maciocia, 2013).

A study by Chen et al. (2019) suggested that LA holds promise as an intervention for alleviating insomnia by potentially reducing sleep latency, diminishing nocturnal awakenings, enhancing sleep efficiency, alleviating anxiety, and mitigating daytime drowsiness. Targeting specific acupoints, especially those associated with anxiety and sleep regulation, may contribute to the therapeutic effects of LA in addressing insomnia.

In conclusion, laser acupuncture offers a safe, effective, and non-invasive approach to treating insomnia, aligning with the principles of traditional Chinese medicine. Further research is needed to explore its mechanisms of action and optimise treatment protocols for improved clinical outcomes.

2.8 Mechanism of Action of Laser Acupuncture

The mechanism underlying the action of laser acupuncture, as well as all forms of acupuncture as a prospective treatment for depression, remains inadequately understood. Numerous mechanisms have been proposed for needle acupuncture, including stimulating significant peripheral nerves, neurovascular bundles, mechanoreceptors, or free nerve endings. However, no specific neuroanatomical structure or pathway has been definitively identified as mediating the therapeutic effect (Napadow et al., 2008). Recent focus has shifted towards the loose connective tissue within intermuscular and intramuscular spaces, as numerous meridians and acupuncture points coincide with this anatomical feature (Langevin & Yandow, 2002).

In acupuncture, needles are inserted at specific acupoints and can be manually stimulated through gentle twisting or up-and-down movements by the practitioner. The depth of needle penetration is also adjusted based on the treatment plan. Patients may experience sensations of De Qi, which include feelings of pressure, warmth, or tingling in the superficial layers of the skin. Several theories have been proposed to explain the mechanisms behind acupuncture's effectiveness, including the gate-control theory of pain and the endorphin and neurotransmitter model (Chon & Lee, 2013). Indeed, another theory posits that acupuncture modulates the transmission of pain signals and influences the release of endogenous endorphins and neurotransmitters, leading to physiological changes in the body (Chon & Lee, 2013).

One notable distinction between needle and laser acupuncture (LA) is that LA does not involve the physical penetration of the skin. Despite our increasing understanding of LA, the precise mechanism of nonthermal, low-dose laser irradiation stimulates acupoints remains to be determined. It is plausible that the mechanism of LA operates independently from our current understanding of acupuncture. Current theories suggest that low-dose laser therapy (LLLT) could

modulate inflammation, alleviate pain, and facilitate tissue repair, provided appropriate irradiation parameters are applied (Chung et al., 2013).

Variables in laser acupuncture (LA) can influence the extent of physiological response. The skin's structure is multilayered, inhomogeneous, and anisotropic, which makes its optical properties complex. Additionally, scattering and absorption cause incident light to generally decrease in an exponential and wavelength-dependent manner as it passes through the skin. Moreover, power output and energy doses can affect light penetration and scattering (Whittaker, 2004). Moreover, variations among individuals in terms of skin pigment, thickness, and anatomical location on the body result in inconsistent light penetration and pose challenges for standardisation. Compounds such as haemoglobin and melanin can absorb ultraviolet radiation and specific wavelengths of visible light (380–625 nm), thereby limiting light transmission through the skin (Whittaker, 2004). In contrast, visible red light and infrared radiation (625–1000 nm) are absorbed to a lesser extent by skin tissues and are, therefore, most commonly utilised in laser acupuncture (LA) treatments (Anderson & Parrish, 1981; Wan et al., 1981).

Indeed, different wavelengths, power outputs, and energy doses significantly impact light penetration and scattering in laser acupuncture (LA) treatments. Due to the numerous confounding variables inherent to LA, achieving precise and objective evaluation remains a challenge.

2.9 Characteristics of Laser

Lasers with an output exceeding 500mW, which are employed for heating and direct tissue effects (such as tissue coagulation in surgical applications), are categorised as Class 4 "hard" lasers (Chon et al., 2019). In contrast, lasers utilised for acupuncture applications typically have a power output ranging from 5 to 499mW and are categorised as Class 3b "soft" lasers.

Energy transmission through the skin is further influenced by various skin structures' absorption of light energy. Light wavelengths ranging from 650 to 900 nm exhibit the highest penetration through the skin. Shorter wavelengths are absorbed by melanin and haemoglobin, while wavelengths longer than 900 nm are absorbed by water. With a well-focused laser beam, red wavelengths (around 648 nm) can penetrate approximately 2–4 cm beneath the skin surface, whereas infrared wavelengths (around 810 nm) can penetrate up to 6 cm (Romberg, 2001; Weber et al., 2007).

2.10 Safety of Laser Acupuncture

Unlike needle acupuncture, laser acupuncture (LA) is non-invasive, making it a more feasible treatment option for young and elderly patients, as well as those who may have a fear of needles (Radmayr et al., 2001). Typically, patients do not experience any sensations when a low-level laser is used during laser acupuncture treatments (Quah-Smith et al., 2010). Although uncommon, some patients have reported experiencing a tingling or "light touch" sensation during laser acupuncture treatments (Quah-Smith et al., 2013). Adverse effects such as transient dizziness, headaches, and fatigue have been reported with laser acupuncture (Quah-Smith et al., 2013). The non-invasive nature of laser acupuncture also minimises the risk of adverse events, such as organ puncture, infection, or bleeding complications, which are associated with needle acupuncture (Chow et al., 2009). Laser acupuncture also facilitates stimulating areas that may be difficult or uncomfortable to needle, such as auricular acupoints (Round et al., 2013). A multichannel laser system can treat multiple acupoints simultaneously, similar to metal-needle acupuncture treatment (Weber et al., 2007).

Due to the potential risk of severe eye damage from the irradiation of Class 3b lasers used in laser acupuncture (LA), both patients and providers must wear protective eyewear during treatment. Additionally, several precautions should be taken, including avoiding irradiation directed towards the foetus in pregnant patients, refraining from irradiating the heart region in patients with cardiac conditions and

steering clear of haemorrhagic areas and the gonads. Furthermore, the epiphyseal line in children should be avoided, and LA treatment should not be administered to children under the age of 2. Direct irradiation of tumours should also be avoided as it may potentially stimulate their growth (Uemoto et al., 2013).

Laser acupuncture (LA) offers numerous advantages, making it an appealing treatment option for specific patient populations. It may be preferred by paediatric and geriatric patients, as well as individuals with needle phobias since it does not involve skin penetration and is associated with minimal sensation and reduced risk of infection, trauma, and bleeding complications. These characteristics render LA more feasible for patients with severe comorbid conditions, hospitalised individuals, and those at higher risk of complications such as bleeding and infection. Moreover, LA treatments typically have shorter durations and multiple acupoints can be targeted within finite treatment times.

A trial done by Lin et al. (2019) reported that the impact of laser acupuncture (LA) treatment on objective sleep maintenance and architecture was distinctly observed through actigraphy. The study spanned a longer duration, involved skilled acupuncturists, and targeted crucial acupoints along the Governor's vessel. The Governor vessel oversees all yang meridians and plays a pivotal role in regulating the balance of yin-yang in the body, a process intricately linked to brain function (Lin et al., 2019). Laser acupuncture (LA) has the potential to soothe nerves, induce relaxation, and alleviate depressive symptoms among patients (Zheng et al., 2019). There have been no comparable large-scale multicentre randomised clinical trials examining the effects of laser acupuncture (LA) in managing concurrent depression and insomnia. This meticulously crafted and rigorously executed trial furnishes crucial clinical evidence concerning the role and significance of LA as an alternative therapy for addressing both insomnia and depressive symptoms.

2.11 Therapeutic Mechanisms of Acupuncture for Insomnia

Many research findings indicate that acupuncture is both safe and effective for insomnia treatment, boasting lasting results and substantial patient adherence. This method is extensively applied in clinical settings (Liu et al., 2020; Mysliwiec et al., 2020). However, a majority of these studies primarily examine the therapeutic efficacy of acupuncture for insomnia, with minimal exploration of its underlying mechanisms. Several pieces of research have determined that acupuncture can notably enhance sleep quality and reduce symptoms of depressive disorders (He et al., 2019). The hypothalamic-pituitary-adrenal (HPA) axis, seen as the core connection between insomnia and depressive disorders onset, has emerged as a focal point in sleep medicine research. Elements like 5-hydroxytryptamine (5-HT), corticosterone (CORT), and other stress-related hormones play roles in the onset of insomnia and its associated depressive disorders (Liu et al., 2021).

2.12 5-hydroxytryptamine (5-HT)

5-HT, a monoamine neurotransmitter, is predominantly found in blood platelets and the central nervous system (CNS) of both animals and humans. It plays a significant role in regulating mood, feelings, and well-being (Harmer, 2008). In the 1960s, the hypothesis linking monoamine-serotonin to depressive disorders emerged, positing that a deficiency in monoamines, such as 5-HT, in the brain might lead to depressive disorders (Hirschfeld, 2000; Cowen, 2008; Albert et al., 2012). As a result, serotonin 5HT, as a powerful neurotransmitter, has its concentrations in the CNS intricately tied to mood-related conditions, notably depressive disorders (Ciarleglio et al., 2011).

2.13 Corticosterone (CORT)

On the other hand, CORT is an adrenal-derived steroid that plays a pivotal role in stress reactions (Ciarleglio et al., 2011). Moreover, CORT has been observed to influence depressive disorder-like tendencies in rat models (Kalynchuk et al., 2004). Research indicates that individuals with insomnia and depressive disorders have

notably elevated serum CORT levels compared to healthy people, whereas their 5-HT levels are considerably reduced (Owens et al., 2014). CORT stands as a standalone risk element for the development of moderate to severe depressive disorders in those with insomnia. Conversely, 5-HT acts as a safeguarding agent. This implies that clinically measuring the levels of 5-HT and CORT can offer valuable insights for assessing the severity of depressive disorders in people with insomnia (Liu et al., 2021).

Methods:

Research design

This is a three-arm, double-blind, randomised controlled trial with an expected duration of three years. The study site to recruit subjects is at the Second Affiliated Hospital of Xinxiang Medical University, Henan, China.

Study area

This study will be carried out at Second Affiliated Hospital of Xinxiang Medical University, Henan, China.

Study population

This study will recruit inpatients diagnosed with insomnia associated with major depressive disorder. Subject recruitment will take place at the Second Affiliated Hospital of Xinxiang Medical College. A pamphlet will be distributed to all potential patients with information about the study project, the intervention protocol, the duration of the study, sample collection, and a feedback form so that the research team can identify patients who are interested in participating in the study. Patients will be selected if they meet all the inclusion criteria and do not have any exclusion criteria.

Subject criteria

a) Inclusion criteria:

- Inpatients with a diagnosis of major depressive disorder (diagnosed according to the relevant diagnostic criteria of the Diagnostic and Statistical Manual of Mental Disorders-V).
- Male or female, 18 to 60 years of age.
- Participants complaining of insomnia at initial screening.
- PSQI score over 7.
- HAMD score between 20 and 35.
- No use of hypnotic medication or acupuncture treatment within the last month.
- No cognitive or communication disorders.
- Willingness to accept random group assignment and sign an informed consent form.
- Those who have been on stable dose of antidepressant for at least the most recent two weeks (SSRI dosage equivalent to escitalopram at 10mg to 20mg/day, SNRI dosage equivalent to venlafaxine at 150mg to 225mg/day, and mirtazapine dosage at 30mg to 45mg ON) and willing to maintain on the same dosage throughout the study.

b) Exclusion criteria:

- Individuals with a marked tendency towards suicide, as assessed by a specialist;
- Individuals previously diagnosed with schizophrenia, bipolar disorder, or other psychiatric disorders;
- Individuals with severe alcohol or drug abuse issues;

- Individuals with liver or kidney dysfunction, or with uncontrollable tumors or significant cerebrovascular diseases;

- Women who are pregnant or breastfeeding;

- Individuals likely to have poor compliance or who are fearful of acupuncture treatment.

Sample size estimation

The calculation of the sample size will be based on an open study of acupuncture for elderly patients with depressive disorders and sleep disorders (Zuppa et al., 2015) . In this reference study, the designer used PSQI as the main assessment indicator and recruited 24 patients in each group to receive either real or sham acupuncture treatment. The baseline PSQI score of the acupuncture group was 8.04±4.0, which significantly decreased (-53.23%; p<0.01) after 10 treatment sessions. We will consider a 20% dropout rate and determine the sample size for each group to be 30 participants. Therefore, with a 1:1:1 allocation to each group, a total of 90 participants will ultimately be recruited.

Sampling method and subject recruitment

Initially, recruitment posters will be displayed on the notice boards of the 2nd Affiliated Hospital, XXMU, China, as well as in the Department of Psychiatry at the same institution. Interested individuals will approach the Department of Psychiatry, XXMU, to undergo screening to determine their eligibility for the study. Eligible candidates will be provided with information about the study's objectives and procedures, the potential benefits and risks of participation, their rights to withdraw at any time, the anonymity of the data collected, data storage methods, and any incentives offered. This information will be thoroughly explained before they are asked to sign the informed consent form.

Randomization:

To maintain impartial allocation and keep group designations confidential, researchers in this study will employ the random number table envelope method for concealment, a straightforward randomisation approach. In essence, SPSS22.0 software will produce random digits, which will be noted on cards. These cards will then be secured in non-transparent envelopes and stored by an appointed individual. Once a participant agrees to join the trial, they'll choose an envelope with a number card based on their enrolment sequence. Prior to any treatment, the doctor will receive and unseal the envelope to uncover the random number, determining the treatment group assignment.

Blinding

This trial will be double blinded. Individuals will be allocated according to proper randomization, and the envelope will be drawn at the time of enrollment to obtain group information. The treatment will take place in an enclosed space, with participants wearing a blindfold, and the effect will be assessed after six weeks using the Bang Blinding Index.

Three groups will be included: LLA, SLA (simulating laser acupuncture without irradiation), and SSRIs. LLA and SLA will have the same 20-minute, 5-times-weekly, 6-week treatment schedule. All groups will adhere to the conventional antidepressant treatment.

The research team (statistical, evaluation, and data analysts) will be blinded to group assignments. Only acupuncturists who are not involved in the research will know the groups and will be prohibited from data collection and analysis. Data collection will be conducted by independent professionals unaware of group assignment and hypotheses. Data analysis will follow a pre-set plan by statisticians uninvolved in the research. .

Data collection and research tool

Intervention Methods

The purpose of this study was to evaluate the efficacy and safety of low-dose laser acupuncture (LLA) for the treatment of insomnia and depressive symptoms. Participants in both the LLA and SLA groups will engage in treatment sessions for 5 consecutive days per week, once daily, with a two-day break each week, over a course of 6 weeks. Each session will last 20 minutes. Prior to every session, participants will prepare by cleansing their skin and wearing an eye mask, while reclining in a consistent room temperature maintained at a minimum of 25°C.

Treatments will be administered by a licensed acupuncturist with at least three years of clinical experience, who has also received standardized training in the specified treatment protocols. The acupoints targeted for both LLA and SLA include Baihui (GV20), Yintang (GV29), Anmian (EX-HN22), Shenmen (HT7), Neiguan (PC6), Sanyinjiao (SP6), and Taichong (LR3), with detailed description of the location of each acupoint is illustrated in Figure 2 of the manuscript .

a) LLA Group Procedures:

In the LLA group, participants will receive treatments using the xS-998D06 semiconductor laser acupoint therapeutic device from Nanjing Xiaosong Medical Instruments. This device, which operates at a wavelength of 650 nm and an output power of 2.3 mW with time average 1.15 mW, each 20-minute session will involve the use of medical tape to

secure the instrument's probe to the designated acupoints. The specific acupoints are detailed in Table 1.

b) SLA Group Procedures:

For the SLA group, the procedure at the same acupoints will be the same as for the LLA group. However, the instrument probe will be fixed to the acupoint but emit a dummy laser, thus acting as a sham treatment to simulate the laser acupuncture experience without the actual laser being emitted.

c) Control Group Setup:

The control group, termed the medication group, will continue to provide a basis for comparison by exclusively using SSRIs, allowing for an evaluation of the specific impacts of LLA in contrast to a non-intervention approach.

d) Medication Use Across All Groups:

All participants will maintain their usual antidepressant regimen and are required to document any changes in their medication, especially if there is a reduction in dosage, which must be approved after psychiatric consultation. In cases of acute insomnia, participants may take Eszopiclone tablets (1mg/tablet) at a controlled dosage of 0.5-1mg, limited to once per night before bedtime. The use of additional sedative hypnotics is generally discouraged; however, any emergency use of such medications must be immediately recorded in the medication log, detailing the date, time, and exact dosage.

Table 1: Acupoints and therapy protocol in the three groups.

**Acupoints**	**LLA group**	**SLA group**	**Control group**
Baihui (GV20)	O	O	×
Yintang (GV29)	O	O	×
Anmian (EX-HN22)	√	√	×
Shenmen (HT7)	√	√	×

Neiguan (PC6)	√	√	×
Sanyinjiao (SP6)	√	√	×
Taichong (LR3)	√	√	×

Note. √: bilateral; ×: non-use; O: single acupoint.

Assessment methods

The study will implement a comprehensive assessment strategy to evaluate the effects of the interventions on sleep quality, depressive symptoms, insomnia severity, anxiety levels, and biological markers. These assessments will occur across four distinct timeframes: baseline (Week-0), immediately post-treatment period (Week 6), and in the post-treatment follow-up period (Week 12 after completion of treatment). This study follows the Standard Protocol Items: Recommendations for Intervention Trials (SPIRIT) guideline for clinical trial.

Instruments and Measures

a) The Pittsburgh Sleep Quality Index (PSQI) is utilized to evaluate the quality of sleep over the preceding month(Buysse et al., 1989). It is comprised of 19 items that collectively contribute to seven component scores, culminating in a global score ranging from 0 to 21. Higher scores on this scale are indicative of poorer sleep quality, with a global score exceeding 5 signalling the presence of significant sleep disturbances. This instrument serves as a comprehensive measure to ascertain the extent of sleep-related issues among participants, providing a quantifiable means to assess the impact of interventions on sleep quality.

b) The Hamilton Depression Rating Scale (HAMD) is a detailed tool designed to assess the severity of depressive symptoms using a 24-item scale. It evaluates a wide range of symptoms including mood disturbances, anxiety, sleep disorders, cognitive impairment, and more. Each symptom is rated on a scale from 0 to 4, with higher scores indicating greater severity. The HAMD is crucial for clinical assessments,

helping to monitor changes in depression severity and the effectiveness of treatments over time(Broen et al., 2015).

c) The Insomnia Severity Index (ISI) is a diagnostic tool aimed at measuring the severity of insomnia and its consequential impact on daily life. It consists of seven questions, with the total scores ranging from 0 to 28. The scoring system is designed such that higher scores represent more severe levels of insomnia. This index is instrumental in evaluating both the nature and intensity of insomnia symptoms, facilitating the identification of individuals who may benefit from further clinical intervention or those who require adjustments in their current treatment plans(Morin et al., 2011).

d) The Self-Rating Anxiety Scale (SAS) is crafted to assess the severity of anxiety symptoms, featuring 20 items each scored from 1 to 4. The cumulative scores range between 20 and 80, where higher scores are indicative of elevated anxiety levels. This scale meticulously captures both psychological and somatic dimensions of anxiety, providing a comprehensive overview of an individual's anxiety experience. As such, the SAS serves as a valuable tool in both clinical and research settings, aiding in the identification of anxiety levels and the evaluation of treatment efficacy(Zung, 1971).

e) Actigraphy utilizes wrist-worn devices to infer sleep and wakefulness periods based on detected movements, providing data on sleep patterns, quality, and duration. This method offers objective insights into sleep behaviors over time, serving as a valuable tool in sleep research and clinical assessments. By capturing continuous movement data, actigraphy allows for the detailed analysis of sleep cycles and disturbances, facilitating the understanding and management of sleep disorders(Ancoli-Israel et al., 2003).

f) The assessment of serum 5-HT and CORT levels involves participants undergoing a fasting blood draw at 8 A.M., both before and after treatment, while being advised to refrain from strenuous activities prior to the procedure. The levels of these

markers will be determined using the enzyme-linked immunosorbent assay (ELISA) technique. Following the collection, blood samples will be centrifuged and then stored at -80°C, preparing them for subsequent analysis. This procedure is crucial for evaluating the biological impact of treatments on these neurotransmitter and hormone levels, providing a biochemical insight into the physiological changes that may accompany therapeutic interventions.

Study flowchart

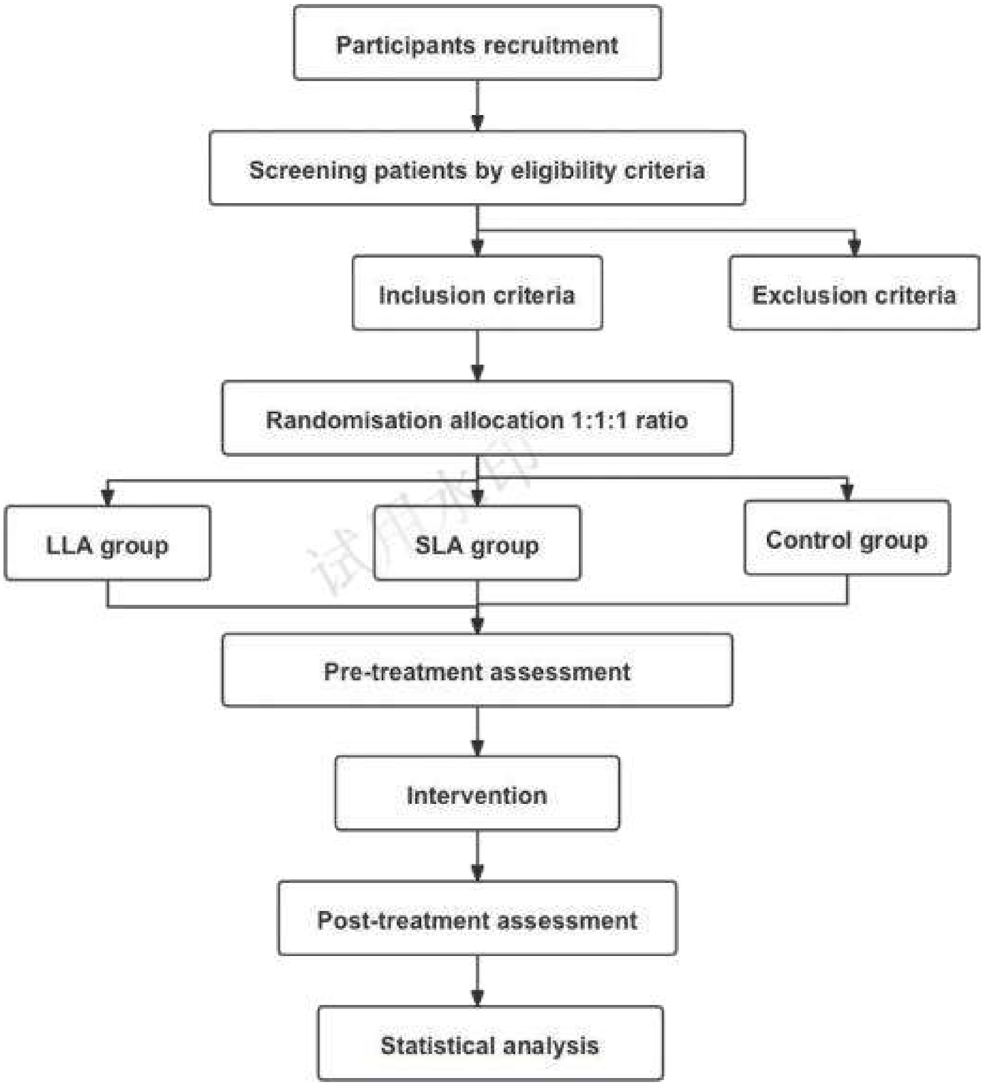

Figure 2: The flowchart of this trial.

Data analysis

All data analysis will be performed using the SPSS version 28. Data will be presented as mean ± standard deviation for normally distributed variables, or median with interquartile range for non-normally distributed variables. Frequencies and percentages will handle categorical data.

Mixed-Model ANOVA will analyze the primary outcome, changes in the Pittsburgh Sleep Quality Index (PSQI) scores, sleep efficiency (SE), total sleep time (TST), number of awakenings, and Insomnia Severity Index (ISI) following the intention-to-treat analysis whereby the interaction of the between-subject factor (the intervention groups) and the within-subject factor (the time points) will be assessed. Effect size will be evaluated with partial Eta square. Any significant interaction will be followed by pairwise comparison of the between-subject factor (between the intervention groups) and the within-subject factor (within the time points) to evaluate the difference in the rate of change in the primary outcome between the intervention groups (LLA, SLA and control groups) across the three time points (baseline, immediate post-treatment and 12 weeks post-treatment). This will achieve the primary objective. The evaluation of the secondary outcomes of severity of depression and anxiety symptoms and the serum levels of cortisol and serotonin will also be carried out similarly to the primary outcome. This will achieve secondary objectives (1) and (2).

To attain secondary objective (3), repeated measure ANOVA is used to compare the severity of insomnia between the LLA + SSRI, LLA + SNRI and LLA + mirtazapine subgroups across the three time points (baseline, immediate post-treatment and 12 weeks post-treatment). Again, effect size will be computed with partial eta square.

To achieve secondary objective (4), the dropout rate due to adverse effects between the LLA, SLA and control groups during the immediate post-treatment and 12 weeks post-treatment are compared using Pearson's chi square test or Fisher's exact test (if one cell or more is less than 5).

Handling of missing data: A tiered approach will address missing data. If missing data constitute less than 5% of the dataset, they will be ignored under the assumption that the absence is random and negligible. For 5–40% missing data, multiple imputation using chained equations will be performed in Stata 15, assuming data are missing at random. If

more than 40% of data are missing or data are suspected to be non-randomly missing, only available data will be analyzed, with limitations discussed in publications.

Expected result(s)

Table 6: Socio-demographic and clinical characteristics

Variables	LLA group		SLA group		Treatment-as-usual control group	
	Frequency (n)	Percentage (%)	Frequency (n)	Percentage (%)	Frequency (n)	Percentage (%)
Age*						
Race:<br>Han Chinese<br>Non-Han Chinese						
Religion:<br>No religion<br>Buddhist/Confucianist/Taoist<br>Other religions						
Marital status:<br>Married<br>Single<br>Divorce/separated/widower						
Education:<br>Up to primary education or lower<br>Up to secondary education<br>Up to tertiary education						
Employment:<br>Employed<br>Unemployed/student<br>Retired						

Monthly household income:<br>< RMB 5000<br>RMB 5000 to RM 10000<br>> RMB 10000						
History of pre-existing medical illness:<br>Yes<br>No						
History of regular medication intake:<br>Yes<br>No						
Duration of insomnia*						
Duration of depression*						
Previous treatment:<br>Medication/Acupuncture/Psychotherapy/Other Treatments						
Medication use: Sedatives/Antidepressants						

* in mean and standard deviation

Table 7: Comparison of the changes in the Pittsburgh Sleep Quality Index (PSQI), Actigraphy, HAMD (Hamilton Depression Rating Scale), SAS (Self-Rating Anxiety Scale), ISI (Insomnia Severity Index), Serum 5-HT (5-Hydroxytryptamine), Serum CORT (Cortisol) between those in the virtual reality exposure therapy group, acceptance and commitment group, and treatment-as-usual group and across four timepoints.

**Variables**	**Time 1 (pre-intervention)**			**Time 2 (4 weeks)**			**Time 3 (6 weeks)**			**Time 3 (18 weeks) (follow-up)**			**p-value**	**Effect size**
	LLA	SLA	Con	LLA	SLA	Con	LLA	SLA	Con	LLA	SLA	Con		
PSQI														
Actigraphy										-	-	-		
HAMD														
SAS														
ISI														
Serum 5-HT										-	-	-		
Serum CORT										-	-	-		

* statistical significance at $p < 0.05$, LLA = low-dose laser acupuncture group, SLA = sham laser acupuncture group, and Con = treatment-as-usual control group.

Budget proposal (from personal funding):

**Vote**<br>*Vot*	**Budget Details & Justification**<br>*Perincian Bajet & Justifikasi*	**Amount requested by researcher (RM)**<br>*Amaun yang dipohon oleh penyelidik (RM)*		
		**Year 1**<br>*Tahun 1*	**Year 2**<br>*Tahun 2*	**Year 3**<br>*Tahun 3*
**11000**<br>Salary and wages<br>*Gaji dan upah*				
**21000**<br>Travelling expenses<br>and subsistence<br>*Perbelanjaan Perjalanan dan Sara Hidup*				
**23000**<br>Communication and Utilities (Phone, Fax, Postage etc)<br>*Perhubungan dan Utiliti*<br>*(Tel,Faks,Pos, dll)*				
**24000**<br>Rental<br>*Sewaan*				
**27000**<br>Research Materials & Supplies (Includes Alcohol Swabs, Tape, Disposables, etc.)<br>*Bekalan & Bahan-bahan Lain (termasuk Haiwan, Pokok dan Bahan Pakai Habis)*	Stationary:<br>• A4 Paper (6 rims) @ RM12 per rim: RM12 x 6 rims= RM 72	36	36	
**28000**				

Maintenance and Minor Repair Services<br>*Penyelenggaraan dan Pembaikan Kecil*				
**29000**<br>Professional services & other services (printing & hospitality, honorarium, conference fee, journals page charges)<br>*Perkhidmatan ikhtisas dan perkhidmatan lain-lain (percetakan hospitaliti, honorarium, yuran persidangan & penerbitan)*	(1) Printing:<br>Photostat copy of questionnaires: at RM0.10 per page x 4 pages x 90 subjects = RM 36 per assessment x 4 assessments = RM 144<br>(2) Honorarium of subjects:<br>RM 20 per subject x 90 subjects x 4 assessments =<br>RM 7,200	144<br><br>7,200		
**35000 (≥ RM1,000)**<br>Equipment[Please provide quotation]<br>*Peralatan [Sila berikan sebutharga]*				
**TOTAL AMOUNT**		7,380	36	
**GRAND AMOUNT**		**7,416 (from personal funding)**		

Ethical consideration(s) [if applicable]:

1. Subject vulnerability

Some questions asked during the study might disturb some subjects. Should any subjects develop mental disturbances while participating in the study, they will be recommended for referrals to Henan Mental Hospital, Henan, China (the nearest psychiatric service available). Additionally, before referral, rescue medication such as benzodiazepines will be administered to calm the patients, if necessary.

Some participants might have adverse reactions (such as skin rash, localized discomfort, etc.) to laser acupuncture treatment. A research team member (a PhD student who is also a medical doctor) will be responsible for managing participants who present with such adverse effects. If the adverse effect persists, participants will then be referred to the XXMU Dermatology Department or another department (depending on the specific adverse reaction) for further examination and treatment. Participants may withdraw from the study should an adverse event occur. An adverse event (AE) is any untoward medical occurrence in a subject administered a trial intervention that does not necessarily have a causal relationship with this treatment. An AE can be any unfavorable and unintended sign, symptom, or disease temporarily associated with the use of investigational intervention, whether or not related to the investigational intervention. Subjects will be issued with a study card with the contact details of the research team, and they are encouraged to maintain close contact by phone, to report any AE occurring in subjects. In the case of an adverse event (AE), the event is reported in the adverse event section in the Case Report Form (CRF), and a serious adverse event report is filled out if necessary (Figure 5). The details to be reported include the name of the event, the date of onset and date of recovery, severity, relationship to the study treatment, measures taken regarding study treatment, treatment of the adverse event, and the outcome of the event (resolved/ongoing). Some reasons that may lead to subjects withdrawing from the study in cases of AE are:

- Presence of adverse reactions not related to the study, but subjects feel uncomfortable continuing in this study.

- Presence of adverse reactions that may be related to the study, such as unusual illnesses that started upon this intervention.

- Unusual changes in behavior, temperament, routine, etc., of subjects, which started upon this intervention.

- Any suspected or unexpected adverse events that are not consistent with the general acceptance of laser acupuncture treatment. Generally, laser acupuncture should not exhibit any side effects or health detrimental effects.

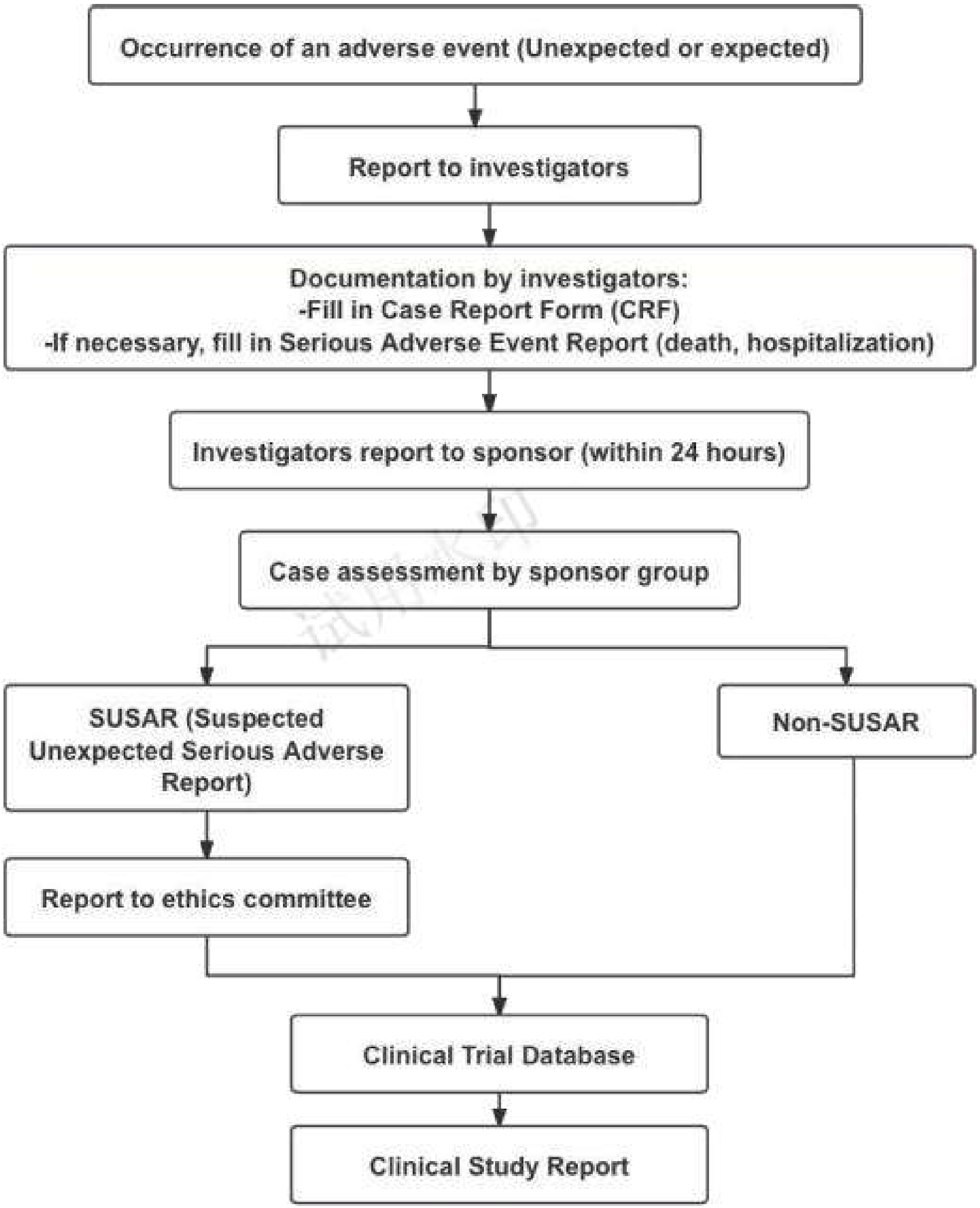

Figure 4: Flow chart of adverse event report.

2. Declaration of absence of conflict of interest

There is no conflict of interest for the research team.

3. Privacy and confidentiality

Subjects' personal identifiable information will not be elicited and they are assured of their participation anonymity. Each subject will be given research number eg

RCT001, etc. The completed questionnaires will be kept in the Department of Psychiatry, 2nd Affiliated Hospital, XXMU, while the data which entered into the document files in the computer will be kept by the primary investigator in USM after completion of the study. All the documents involved in assessment of all subjects including subject's personal information (socio-demographic, substance history, symptomatology, and response to questionnaires) are kept in document files and locked in a cabinet with the key kept by the primary investigator. Only the primary investigator and co-researchers are allowed to access the files for data analysis and for publication purposes. The files will be kept for duration of 2 years after completion of the study and last publication of the research findings and then it will be destroyed completely. However, the information will be kept confidential by the researchers and will not be made publicly available unless disclosure is required by law.

4. Community sensitivities and benefits

The community will benefit from the findings of this study as we provide scientific evidence on the effectiveness of laser acupuncture treatment for depression with concurrent insomnia. This will enable clinicians who manage patients with depression and insomnia to integrate this intervention into their comprehensive management plans. The study aims to enhance the screening of patients with these conditions, introduce an additional therapeutic approach for the rehabilitation of affected individuals, and improve relationships between patients and their family members.

Participants may gain from the study as we offer insights into their mental health status. Any participants identified with depression and anxiety disorders will be recommended for further treatment and follow-up at the Henan Mental Hospital, Henan, China. Additionally, participants undergoing laser acupuncture are expected to benefit from this treatment, which is anticipated to be effective in addressing symptoms of depression and insomnia. This effective intervention for achieving

remission from these conditions is also expected to assist participants in managing their lives, securing permanent employment, and enhancing their family relationships.

5. Honorarium and incentives

As a token of appreciation, respondents were paid an honorarium of MYR 20 per assessment in recognition of their willingness to participate in the study and the time they spent. Thus, each patient participated in the study for only 25 minutes per session, for a total participation time of 10 hours.

6. Monitoring of clinical trial

Any modifications to the study protocol (e.g., inclusion and exclusion criteria of study subjects, specific study operational procedures, outcome measures for each indicator, intended interventions, and subsequent data analysis methods) will be communicated in writing to both the Ethics Committee of the 2nd Affiliated Hospital, XMU, China and the Human Research Ethics Committee of USM.

The principal investigator will oversee the trial and conduct the study at the 2nd Affiliated Hospital, XMU, collaborating closely with the research team, participating in participant recruitment, supervising the consent process and controlling the intervention programme. A trial monitoring committee, chaired by the principal investigator, has been established. This committee convenes weekly to oversee the study's daily operations, review the trial process, and prepare reports for submission to the Ethics Committee of the 2nd Affiliated Hospital, XMU and the Human Research Ethics Committee of USM. Data monitoring and auditing of the trial will be undertaken by qualified personnel not associated with the funding of the research.

When one or more subjects in the randomized intervention group (LLA or SLA) experience an unexpected and serious AE, an interim analysis will be performed to evaluate the circumstances. Should the investigation findings indicate a safety issue associated with the intervention (LLA or SLA), the study Oversight Committee will

take steps to prematurely conclude the study. The serious AEs that were cited as the basis for early termination of the trial included situations such as suicidal ideation, life-threatening self-injurious behaviors, and hospitalization as a result of adverse psychological events associated with the intervention.

Access to the research documents is granted only to the corresponding author and co-authors of the study for the sole purpose of data analysis or writing for publication. The results of this study will be disseminated via publishing in peer-reviewed publications and presented at internationally conferences and seminars. Corresponding authors of this study will be the principal investigators and authorship will follow the recommendations of the International Committee of Medical Journal Editors.

All researchers who are currently conducting this study formally acknowledge that they have no financial interests or other conflicts of interest that are associated with the conduct or progression of this study.

7. Other ethical review board approval [if applicable]

We have obtained approval for the study by the Human Research Ethics Committee of Xinxiang Medical University, Henan, China (code: XYEFYLL-(科研)-2024-36).

References

Albert, P. R., Benkelfat, C., & Descarries, L. (2012). The neurobiology of depression-revisiting the serotonin hypothesis. I. cellular and molecular mechanisms. Philosophical Transactions of the Royal Society B: Biological Sciences, 367(1601), 2378–2381.

Ancoli-Israel, S., Cole, R., Alessi, C., Chambers, M., Moorcroft, W., & Pollak, C. P. (2003). The role of actigraphy in the study of sleep and circadian rhythms. Sleep, 26(3), 342-392.

Anderson, R. R., & Parrish, J. A. (1981). The optics of human skin. Journal of Investigative Dermatology, 77(1), 13–19.

Andrade, L., Caraveo-Anduaga, J. J., Berglund, P., Bijl, R. V., De Graaf, R., Vollebergh, W., Dragomirecka, E., et al. (2003). The epidemiology of major depressive episodes: Results from the International Consortium of Psychiatric Epidemiology (ICPE) Surveys. International Journal of Methods in Psychiatric Research, 12(1), 3–21.

Armitage, R. (2007). Sleep and circadian rhythms in mood disorders. Acta Psychiatrica Scandinavica, 115(SUPPL. 433), 104–115.

Baglioni, C., Battagliese, G., Feige, B., Spiegelhalder, K., Nissen, C., Voderholzer, U., Lombardo, C., & Riemann, D. (2011). Insomnia as a predictor of depression: A meta-analytic evaluation of longitudinal epidemiological studies. Journal of Affective Disorders, 135(1-3), 10-19.

Bertisch, S. M., Wells, R. E., Smith, M. T., & McCarthy, E. P. (2012). Use of relaxation techniques and complementary and alternative medicine by American adults with insomnia symptoms: Results from a national survey. Journal of Clinical Sleep Medicine, 8(6), 681–691.

Bolge, S. C., Doan, J. F., Kannan, H., & Baran, R. W. (2009). Association of insomnia with quality of life, work productivity, and activity impairment. Quality of Life Research, 18(4), 415–422.

Broen, M. P., Moonen, A. J., Kuijf, M. L., Dujardin, K., Marsh, L., Richard, I., Starkstein, S. E., Martinez–Martin, P., & Leentjens, A. F. (2015). Factor analysis of the Hamilton Depression Rating Scale in Parkinson's disease. Parkinsonism & Related Disorders, 21(2), 142-146.

Buysse, D. J., Reynolds III, C. F., Monk, T. H., Berman, S. R., & Kupfer, D. J. (1989). The Pittsburgh Sleep Quality Index: a new instrument for psychiatric practice and research. Psychiatry Research, 28(2), 193-213.

Cardinali, D. P., Srinivasan, V., Brzezinski, A., & Brown, G. M. (2012). Melatonin and its analogs in insomnia and depression. Journal of Pineal Research, 52(4), 365–375.

Chan, Y. Y., Lo, W. Y., Yang, S. N., Chen, Y. H., & Lin, J. G. (2015). The benefit of combined acupuncture and antidepressant medication for depression: A systematic review and meta-analysis. Journal of Affective Disorders, 176(91), 106–117.

Cheuk, D. K. L., Yeung, W. F., Chung, K. F., & Wong, V. (2007). Acupuncture for insomnia. The Cochrane Database of Systematic Reviews, 18(3), CD005472.

Cheung, J. M. Y., & Wong, M. M. (2011). The effects of insomnia and sleep loss on cardiovascular disease. Sleep Medicine Reviews, 15(4), 222-231.

Cheung, L. M., & Wong, W. S. (2011). The effects of insomnia and internet addiction on depression in Hong Kong Chinese adolescents: An exploratory cross-sectional analysis. Journal of Sleep Research, 20(2), 311–317.

Chien, H. C., Chung, Y. C., Yeh, M. L., & Lee, J. F. (2015). Breathing exercise combined with cognitive behavioural intervention improves sleep quality and heart rate variability in major depression. Journal of Clinical Nursing, 24(21–22), 3206–3214.

Chon, T. Y., Mallory, M. J., Yang, J., Bublitz, S. E., Do, A., & Dorsher, P. T. (2019). Laser Acupuncture: A Concise Review. Medical Acupuncture, 31(3), 164–168.

Chon, T. Y., & Lee, M. C. (2013). Acupuncture. Mayo Clinic Proceedings, 88(10), 1141–1146.

Chow, R. T., Johnson, M. I., Lopes-Martins, R. A., & Bjordal, J. M. (2009). Efficacy of low-level laser therapy in the management of neck pain: a systematic review and meta-analysis of randomised placebo or active-treatment controlled trials. The Lancet, 374(9705), 1897–1908.

Chung, H., Dai, T., Sharma, S. K., Huang, Y.-Y., Carroll, J. D., & Hamblin, M. R. (2013). The Nuts and Bolts of Low-level Laser (Light) Therapy. Annals of Biomedical Engineering, 40(2), 516–533.

Ciarleglio, C. M., Resuehr, H. E. S., & McMahon, D. G. (2011). Interactions of the serotonin and circadian systems: Nature and nurture in rhythms and blues. Neuroscience, 197, 8–16.

Coryell, V. T., Ziegelstein, R. C., Hirt, K., Quain, A., Marine, J. E., & Smith, M. T. (2013). Clinical correlates of insomnia in patients with acute coronary syndrome. International Heart Journal, 54(5), 258–265.

Coryell, W., Fiedorowicz, J., Solomon, D., & Endicott, J. (2013). Prospective assessment of Axis I psychiatric disorders after the onset of a first episode of major depressive disorder: Results from the Iowa 500. Journal of Clinical Psychiatry, 74(4), e293-e300.

Cowen, P. J. (2008). Serotonin and depression: pathophysiological mechanism or marketing myth?. Trends in Pharmacological Sciences, 29(9), 433–436.

Daley, M., Morin, C. M., LeBlanc, M., Grégoire, J. P., Savard, J., & Baillargeon, L. (2009). Insomnia and its relationship to health-care utilization, work absenteeism, productivity and accidents. Sleep Medicine, 10(4), 427–438.

Dement, W. C. (2010). History of Sleep Physiology and Medicine. Principles and Practice of Sleep Medicine: Fifth Edition, Fifth Edition. Elsevier Inc.

Difrancesco, S., Lamers, F., Riese, H., Merikangas, K. R., Beekman, A. T. F., van Hemert, A. M., Schoevers, R. A., et al. (2019). Sleep, circadian rhythm, and physical activity patterns in depressive and anxiety disorders: A 2-week ambulatory assessment study. Depression and Anxiety, 36(10), 975–986.

Dong, B., Chen, Z., Yin, X., Li, D., Ma, J., Yin, P., Cao, Y., et al. (2017). The Efficacy of Acupuncture for Treating Depression-Related Insomnia Compared with a Control Group: A Systematic Review and Meta-Analysis. BioMed Research International, 2017.

Dudek, B., & Koniarek, J. (2000). Relationship between sense of coherence and post-traumatic stress disorder symptoms among firefighters. International Journal of Occupational Medicine and Environmental Health, 13(4), 299–305.

Ellis, P. M., & Smith, D. A. R. (2002). Treating depression: the beyondblue guidelines for treating depression in primary care. Medical Journal of Australia, 176, S77–S83.

Fang, H., Tu, S., Sheng, J., & Shao, A. (2019). Depression in sleep disturbance: A review on a bidirectional relationship, mechanisms and treatment. Journal of Cellular and Molecular Medicine, 23(4), 2324–2332.

Ferrari, A. J., Charlson, F. J., Norman, R. E., Patten, S. B., Freedman, G., Murray, C. J. L., Vos, T., et al. (2013). Burden of Depressive Disorders by Country, Sex, Age, and Year: Findings from the Global Burden of Disease Study 2010. PLoS Medicine, 10(11), e1001547.

Fox, M. R. (1999). The importance of sleep. *Nursing Standard (through 2013)*, *13*(24), 44.

Gamaleya, N. F. (1977). Laser Biomedical Research in the USSR. Laser Applications in Medicine and Biology, 1961, 1–173.

Gibson-Smith, D., Bot, M., Milaneschi, Y., Twisk, J. W., Visser, M., Brouwer, I. a., & Penninx, B. W. J. H. (2015). Understanding and Managing Withdrawal Syndromes After Discontinuation of Antidepressant Drugs. Journal of Clinical Psychiatry, 77(September), 22–27.

Gold, P. W., & Chrousos, G. P. (2002). Organization of the stress system and its dysregulation in melancholic and atypical depression: High vs low CRH/NE states. Molecular Psychiatry, 7(3), 254-275.

Greenberg, P. E., & Birnbaum, H. G. (2005). The Economic Burden of Depression: Societal and Patient Perspectives. Expert Opinion on Pharmacotherapy, 6(3), 33–45.

Haaramo, P., Lallukka, T., Lahelma, E., Hublin, C., & Rahkonen, O. (2014). Insomnia symptoms and subsequent psychotropic medication: A register-linked study with 5-year follow-up. Social Psychiatry and Psychiatric Epidemiology, 49(12), 1993–2002.

Halbreich, U. (2008). Systematic reviews of clinical trials of acupuncture as treatment for depression: How systematic and accurate are they?. CNS Spectrums, 13(4), 293–300.

Harmer, C. J. (2008). Serotonin and emotional processing: Does it help explain antidepressant drug action?. Neuropharmacology. Elsevier Ltd, 55(6), 1023–1028.

He, W., Li, M., Zuo, L., Wang, M., Jiang, L., Shan, H., Han, Xue, et al. (2019). Acupuncture for treatment of insomnia: An overview of systematic reviews. Complementary Therapies in Medicine. Elsevier, 42(November 2018), 407–416.

Hirschfeld, R. M. (2000). History and evolution of the monoamine hypothesis of depression. Journal of Clinical Psychiatry, 61(6), 4–6.

Hu, W. L., Chang, C. H., Hung, Y. C., Tseng, Y. J., Hung, I. L., & Hsu, S. F. (2014). Laser acupuncture therapy in patients with treatment-resistant temporomandibular disorders. PLoS ONE, 9(10).

Huang, Y., Wang, Y., Wang, H., Liu, Z., Yu, X., Yan, J., Yu, Y., et al. (2019). Prevalence of mental disorders in China: a cross-sectional epidemiological study. The Lancet Psychiatry. Elsevier Ltd, 6(3), 211–224.

Huo, Z. J., Guo, J., & Li, D. (2013). Effects of acupuncture with meridian acupoints and three anmian acupoints on insomnia and related depression and anxiety state. Chinese Journal of Integrative Medicine, 19(3), 187–191.

James, S. L., Abate, D., Abate, K. H., Abay,S. M., Abbafati, C., Abbasi, N., Abbastabar, H., et al. (2018). Global, regional, and national incidence, prevalence, and years lived with disability for 354 Diseases and Injuries for 195 countries and territories, 1990-2017: A systematic analysis for the Global Burden of Disease Study 2017. The Lancet, 392(10159), 1789–1858.

Jang, I., Sun, S., & Jeong, M. (2019). Early history of laser acupuncture: who used it first?. Integrative Medicine Research. Korea Institute of Oriental Medicine, 8(2), 129–130.

Jiang, B., Ma, Z., & Zuo, F. (2010). Auricular acupuncture for insomnia:a randomized controlled trial. Zhonghua Liu Xing Bing Xue Za Zhi, 31(12), 1400–1402.

Kalynchuk, L. E., Gregus, A., Boudreau, D., & Perrot-Sinal, T. S. (2004). Corticosterone increases depression-like behavior, with some effects on predator odor-induced defensive behavior, in male and female rats. Behavioral Neuroscience, 118(6), 1365–1377.

Kato, T. (2014). Insomnia symptoms, depressive symptoms, and suicide ideation in japanese white-collar employees. International Journal of Behavioral Medicine, 21(3), 506–510.

Kessler, R. C., Berglund, P., Demler, O., Jin, R., Koretz, D., Merikangas, K. R., Rush, A. J., et al. (2003). The epidemiology of major depressive disorder. Evidence-Based Eye Care, 4(4), 186–187.

Lader, M., Tylee, A., & Donoghue, J. (2009). Withdrawing benzodiazepines in primary care. CNS Drugs, 23(1), 19–34.

Langevin, H. M., & Yandow, J. A. (2002). Relationship of acupuncture points and meridians to connective tissue planes. Anatomical Record, 269(6), 257–265.

Lapierre, S., Boyer, R., Desjardins, S., Dubé, M., Lorrain, D., Préville, M., & Brassard, J. (2012). Daily hassles, physical illness, and sleep problems in older adults with wishes to die. International Psychogeriatrics, 24(2), 243–252.

Lee, S. Y., Baek, Y. H., Park, S. U., Moon, S. K., Park, J. M., Kim, Y. S., & Jung, W. S. (2009). Intradermal acupuncture on Shen-Men and Nei-Kuan acupoints improves insomnia in stroke patients by reducing the sympathetic nervous activity: A randomized clinical trial. American Journal of Chinese Medicine, 37(6), 1013–1021.

Leo, R. J., & Ligot, J. S. A. (2007). A systematic review of randomized controlled trials of acupuncture in the treatment of depression. Journal of Affective Disorders, 97(1–3), 13–22.

Li, X., Zhu, X., Li, R., & Zhu, F. (2022). Efficacy and safety of acupuncture for cervicogenic insomnia: A protocol for a systematic review and meta-analysis. Proceedings - 2022 12th International Conference on Information Technology in Medicine and Education, ITME 2022, (July), 43–47.

Lin, H. T., Lai, C. H., Perng, H. J., Chung, C. H., Wang, C. C., Chen, W. L., & Chien, W. C. (2018). Insomnia as an independent predictor of suicide attempts: A nationwide population-based retrospective cohort study. BMC Psychiatry. BMC Psychiatry, 18(1), 1–11.

Lin, L., Yu, L., Xiang, H., Hu, X., Yuan, X., Zhu, H., Li, H., et al. (2019). Effects of Acupuncture on Behavioral Stereotypies and Brain Dopamine System in Mice as a Model of Tourette Syndrome. Frontiers in Behavioral Neuroscience, 13(October), 1–15.

Lin, Y. F., Liu, Z. D., Ma, W., & Shen, W. D. (2016). Hazards of insomnia and the effects of acupuncture treatment on insomnia. Journal of Integrative Medicine. Journal of Integrative Medicine Editorial Office. E-edition published by Elsevier (Singapore) Pte Ltd. All rights reserved., 14(3), 174–186.

Ling, L., Jiang, X. M., Xue, J. W., Wang, M., & Ke, R. (2008). Clinical study on the visceral differentiation-based acupuncture therapy for insomnia. Journal of

Traditional Chinese Medicine. The Editorial Board of Journal of Traditional Chinese Medicine, 28(4), 270–273.

Litscher, G. (2018). Definition of Laser Acupuncture and All Kinds of Photo Acupuncture. Medicines, 5(4), 117.

Liu, C., Xi, H., Wu, W., Wang, X., Qin, S., Zhao, Y., Zheng, S., et al. (2020). Placebo effect of acupuncture on insomnia: A systematic review and meta-analysis. Annals of Cardiothoracic Surgery, 9(1), 19–29.

Liu, C., Zhao, Y., Qin, S., Wang, X., Jiang, Y., & Wu, W. (2021). Randomized controlled trial of acupuncture for anxiety and depression in patients with chronic insomnia. Annals of Translational Medicine, 9(18), 1426–1426.

Lu, D. P., & Lu, G. P. (2013). A comparison of the clinical effectiveness of various acupuncture points in reducing anxiety to facilitate hypnotic induction. International Journal of Clinical and Experimental Hypnosis, 61(3), 271–281.

Lu, J., Shao, R. H., Jin, S. Y., Hu, L., Tu, Y., & Guo, J. Y. (2017). Acupuncture ameliorates inflammatory response in a chronic unpredictable stress rat model of depression. Brain Research Bulletin, 128, 106–112.

Luo, W.-Z., Zhang, Q.-Z., & Lai, X.-S. (2010). Effect of acupuncture treatment of relieving depression and regulating mind on insomnia accompanied with depressive disorders. Zhongguo Zhen Jiu, 30(11), 899–903.

Maciocia, G. (2013). Diagnosis in Chinese Medicine: A Comprehensive Guide. 1st Editio. Churchill Livingstone, Edinburgh, UK: Elsevier Health Sciences.

MacPherson, H., Sinclair-Lian, N., & Thomas, K. (2006). Patients seeking care from acupuncture practitioners in the UK: A national survey. Complementary Therapies in Medicine, 14(1), 20–30.

Matsuda, R., Kohno, T., Kohsaka, S., Fukuoka, R., Maekawa, Y., Sano, M., Takatsuki, S., et al. (2017). The prevalence of poor sleep quality and its association with depression and anxiety scores in patients admitted for cardiovascular disease: A cross-sectional designed study. International Journal of Cardiology. Elsevier B.V., 228, 977–982.

McCall, W. V., Blocker, J. N., D'Agostino, R., Kimball, J., Boggs, N., Lasater, B., Haskett, R., et al. (2010). Treatment of insomnia in depressed insomniacs: Effects on health-related quality of life, objective and self-reported sleep, and depression. Journal of Clinical Sleep Medicine, 6(4), 322–329.

Medina-Chávez, J. H., Fuentes-Alexandro, S. A., Gil-Palafox, I. B., Adame-Galván, L., Solís-Lam, F., Sánchez-Herrera, L. Y., & Sánchez-Narváez, F. (2014). Clinical practice

guideline. Diagnosis and treatment of insomnia in the elderly. Revista Médica del Instituto Mexicano del Seguro Social, 52(1), 108–119.

Merrigan, J. M., Buysse, D. J., Bird, J. C., & Livingston, E. H. (2013). Insomnia. The Journal of the American Medical Association, 309(7), 733.

Moskvin, S. V., & Agasarov, L. G. (2020). Laser Acupuncture: 35 Years of Successful Application in Russia (Narrative Review). Journal of Lasers in Medical Sciences, 11(4), 381–389.

Mukaino, Y., Park, J., White, A., & Ernst, E. (2005). The effectiveness of acupuncture for depression - A systematic review of randomised controlled trials. Acupuncture in Medicine, 23(2), 70–76.

Murphy, M., & Peterson, M. (2015). Sleep Disturbances in Depression. Sleep Medicine Clinics, 10(1), 17–23.

Mysliwiec, V., Martin, J. L., Ulmer, C. S., Chowdhuri, S., Brock, M. S., Spevak, C., & Sall, J. (2020). The management of chronic insomnia disorder and obstructive sleep apnea: Synopsis of the 2019 U.S. Department of Veterans Affairs and U.S. Department of Defense clinical practice guidelines. Annals of Internal Medicine, 172(5), 325–336.

Nadorff, M. R., Fiske, A., Sperry, J. A., Petts, R., & Gregg, J. J. (2013). Insomnia symptoms, nightmares, and suicidal ideation in older adults. Journals of Gerontology - Series B Psychological Sciences and Social Sciences, 68(2), 145–152.

Napadow, V., Ahn, A., Longhurst, J., Lao, L., Stener-Victorin, E., Harris, R., & Langevin, H. M. (2008). The status and future of acupuncture clinical research. Journal of Alternative and Complementary Medicine, 14(7), 861–869.

Ng, T. K., & Wong, D. F. K. (2018). The efficacy of cognitive behavioral therapy for Chinese people: A meta-analysis. Australian and New Zealand Journal of Psychiatry, 52(7), 620–637.

Nutt, D. J., Wilson, S., & Paterson, L. (2008). Sleep disorders as core symptoms of depression. Dialogues in Clinical Neuroscience, 10(3), 329–336.

Ohayon, M. M. (1997). Prevalence of DSM-IV diagnostic criteria of insomnia: Distinguishing insomnia related to mental disorders from sleep disorders. Journal of Psychiatric Research, 31(3), 333–346.

Ohayon, M. M. (2009). Observation of the Natural Evolution of Insomnia in the American General Population Cohort. Sleep Medicine Clinics, 4(1), 87–92.

Ohayon, M. M., & Roth, T. (2003). Place of chronic insomnia in the course of depressive and anxiety disorders. Journal of Psychiatric Research, 37(1), 9–15.

Okajima, I., Komada, Y., Nomura, T., Nakashima, K., & Inoue, Y. (2012). Insomnia as a Risk for Depression: A Longitudinal Epidemiologic Study on a Japanese Rural Cohort. The Journal of Clinical Psychiatry, 73(3), 377–383.

Orzeł-Gryglewska, J. (2010). Consequences of sleep deprivation. International Journal of Occupational Medicine and Environmental Health, 23(1), 95–114.

Owens, M., Herbert, J., Jones, P. B., Sahakian, B. J., Wilkinson, P. O., Dunn, V. J., Croudace, T. J., et al. (2014). Elevated morning cortisol is a stratified population-level biomarker for major depression in boys only with high depressive symptoms. Proceedings of the National Academy of Sciences of the United States of America, 111(9), 3638–3643.

Pace-Schott, E. F., Germain, A., & Milad, M. R. (2012). Sleep and REM sleep disturbance in the pathophysiology of PTSD: The role of extinction memory. Biology of Mood & Anxiety Disorders, 2(1), 1-17.

Palagini, L., Baglioni, C., Ciapparelli, A., Gemignani, A., & Riemann, D. (2013). REM sleep dysregulation in depression: State of the art. Sleep Medicine Reviews, 17(5), 377-390.

Pigott, H. E., Leventhal, A. M., Alter, G. S., & Boren, J. J. (2010). Efficacy and effectiveness of antidepressants: Current status of research. Psychotherapy and Psychosomatics, 79(5), 267–279.

Punnoose, A. R., Golub, R. M., & Burke, A. E. (2012). Insomnia. The Journal of the American Medical Association, 307(24), 2653.

Quah-Smith, I., Sachdev, P. S., Wen, W., Chen, X., & Williams, M. A. (2010). The brain effects of laser acupuncture in healthy individuals: An fMRI investigation. PLoS ONE, 5(9), 1–5.

Quah-Smith, I., Williams, M. A., Lundeberg, T., Suo, C., & Sachdev, P. (2013). Differential brain effects of laser and needle acupuncture at LR8 using functional MRI. Acupuncture in Medicine, 31(3), 282–289.

Quah-Smith, J. I., Tang, W. M., & Russell, J. (2005). Laser acupuncture for mild to moderate depression in a primary care setting - A randomised controlled trial. Acupuncture in Medicine, 23(3), 103–111.

Radmayr, C., Schlager, A., Studen, M., & Bartsch, G. (2001). Prospective randomized trial using laser acupuncture versus desmopressin in the treatment of nocturnal enuresis. European Urology, 40(2), 201–205.

Romberg, H. (2001). How is (laser)-light working. COMED, 11, 27–33.

Roth, T. (2007). Insomnia: Definition, prevalence, etiology, and consequences. Journal of Clinical Sleep Medicine, 3(5 SUPPL.), 3–6.

Roth, T., & Ancoli-Israel, S. (1999). Daytime consequences and correlates of insomnia in the United States: results of the 1991 National Sleep Foundation Survey. II. Sleep, 22(Suppl 2), S354–S358.

Round, R., Litscher, G., & Bahr, F. (2013). Auricular acupuncture with laser. Evidence-based Complementary and Alternative Medicine, 2013.

Saarni, S. I., Suvisaari, J., Sintonen, H., Pirkola, S., Koskinen, S., Aromaa, A., & Lönnqvist, J. (2007). Impact of psychiatric disorders on health-related quality of life: General population survey. British Journal of Psychiatry, 190(APR.), 326–332.

Salo, P., Oksanen, T., Sivertsen, B., Hall, M., Pentti, J., Virtanen, M., Vahtera, J., & Kivimäki, M. (2012). Sleep disturbances as a predictor of cause-specific work disability and delayed return to work. Sleep, 35(10), 1351-1360.

Salo, P., Sivertsen, B., Oksanen, T., Sjösten, N., Pentti, J., Virtanen, M., Kivimäki, M., et al. (2012). Insomnia symptoms as a predictor of incident treatment for depression: Prospective cohort study of 40,791 men and women. Sleep Medicine. Elsevier B.V., 13(3), 278–284.

Sasai, T., Inoue, Y., Komada, Y., Nomura, T., Matsuura, M., & Matsushima, E. (2010). Effects of insomnia and sleep medication on health-related quality of life. Sleep Medicine. Elsevier B.V., 11(5), 452–457.

Sateia, M. J. (2014). International classification of sleep disorders-third edition highlights and modifications. Chest. The American College of Chest Physicians, 146(5), 1387–1394.

Schutte-Rodin, S. L., Broch, L., Buysee, D., Dorsey, C., & Sateia, M. (2008). Clinical guideline for the evaluation and management of chronic insomnia in adults. Journal of Clinical Sleep Medicine, 4(5), 487–504.

Sivertsen, B., Salo, P., Mykletun, A., Hysing, M., Pallesen, S., Krokstad, S., Nordhus, I. H., et al. (2012). The bidirectional association between depression and insomnia: The HUNT study. Psychosomatic Medicine, 74(7), 758–765.

Smith, C. A., Armour, M., Lee, M. S., Wang, L. Q., & Hay, P. J. (2018). Acupuncture for depression. Cochrane Database of Systematic Reviews, 2018(3).

Spence, D. W., Kayumov, L., Chen, A., Lowe, A., Jain, U., Katzman, M. A., Shen, J., et al. (2004). Acupuncture Increases Nocturnal Melatonin Secretion and Reduces Insomnia and Anxiety: A Preliminary Report. Journal of Neuropsychiatry and Clinical Neurosciences, 16(1), 19–28.

Spira, A. P., Kaufmann, C. N., Kasper, J. D., Ohayon, M. M., Rebok, G. W., Skidmore, E., Parisi, J. M., et al. (2014). Association between insomnia symptoms and functional status in U.S. older adults. Journals of Gerontology, Series, B: Psychological Sciences and Social Sciences, 69(7), S35–S41.

Srisurapanont, M., Likhitsathian, S., Chua, H. C., Udomratn, P., Chang, S., Maneeton, N., Maneeton, B., et al. (2015). Clinical and sociodemographic correlates of severe insomnia in psychotropic drug-free, Asian outpatients with major depressive disorder. Journal of Affective Disorders. Elsevier, 186, 26–31.

Suh, S., Kim, H., Yang, H. C., Cho, E. R., Lee, S. K., & Shin, C. (2013). Longitudinal course of depression scores with and without insomnia in non-depressed individuals: A 6-year follow-up longitudinal study in a Korean cohort. Sleep, 36(3), 369–376.

Sunderajan, P., Gaynes, B. N., Wisniewski, S. R., Miyahara, S., Fava, M., Akingbala, F., DeVeaugh-Geiss, J., et al. (2010). Insomnia in patients with depression: A STARD report. CNS Spectrums, 15(6), 394–404.

Tariq, S. H., & Pulisetty, S. (2008). Pharmacotherapy for Insomnia. Clinics in Geriatric Medicine, 24(1), 93–105.

Taylor, D., Gehrman, P., Dautovich, N., Lichstein, K., & McCrae, C. (2014). Handbook of Insomnia. London, UK: Springer Healthcare.

Uemoto, L., Nascimento De Azevedo, R., Almeida Alfaya, T., Nunes Jardim Reis, R., Depes De Gouvêa, C. V., & Cavalcanti Garcia, M. A. (2013). Myofascial trigger point therapy: Laser therapy and dry needling. Current Pain and Headache Reports, 17(9), 1–6.

Van Wingen, G. A., Van Eijndhoven, P., Tendolkar, I., Buitelaar, J., Verkes, R. J., & Fernández, G. (2011). Neural basis of emotion recognition deficits in first-episode major depression. Psychological Medicine, 41(7), 1397–1405.

Wan, S., Parrish, J. A., Anderson, R. R., & Madden, M. (1981). Transmittance of Nonionizing Radiation in Human Tissues. Photochemistry and Photobiology, 34(6), 679–681.

Wang, L., Wang, R., Yao, Y., Bai, X., & Sheng, G. (2022). The effects of acupuncture on psychological symptoms in patients with insomnia: study protocol for a randomized controlled trial. Trials. Trials, 23(1), 1–7.

Wang, X., Cheng, S., & Xu, H. (2019). Systematic review and meta-analysis of the relationship between sleep disorders and suicidal behaviour in patients with depression. BMC Psychiatry. BMC Psychiatry, 19(1), 1–13.

Weber, M., Fussgänger-May, T., & Wolf, T. (2007). ‘Needles of Light’: A New Therapeutic Approach. Medical Acupuncture, 19(3), 141–150.

Whittaker, P. (2004). Laser acupuncture: Past, present, and future. Lasers in Medical Science, 19(2), 69–80.

Wichniak, A., Wierzbicka, A., Walęcka, M., & Jernajczyk, W. (2017). Effects of Antidepressants on Sleep. Current Psychiatry Reports, 19(9), 1–7.

Winokur, A., Gary, K. A., Rodner, S., Rae-Red, C., Fernando, A. T., & Szuba, M. P. (2001). Depression, sleep physiology, and antidepressant drugs. Depression and Anxiety, 14(1), 19–28.

Wright, K. M., Britt, T. W., Bliese, P. D., Adler, A. B., Picchioni, D., & Moore, D. (2011). Insomnia as predictor versus outcome of PTSD and depression among Iraq combat veterans. Journal of Clinical Psychology, 67(12), 1240–1258.

Depression, W. H. O. (2017). Other common mental disorders: global health estimates. *Geneva: World Health Organization*, *24*.

Yang, D., & Zhai, S.-Q. (2013). The relationship between meridians and mental state discussed from the treatment of depression with acupuncture and moxibustion. Zhongguo Zhen Jiu, 33(4), 317–320.

Yang, Y. H., Lai, J. N., Lee, C. H., Wang, J. Der, & Chen, P. C. (2011). Increased risk of hospitalization related to motor vehicle accidents among people taking zolpidem: A case-crossover study. Journal of Epidemiology, 21(1), 37–43.

Yin, X., Dong, B., Liang, T., Yin, P., Li, X., Lin, X., Zhou, S., et al. (2019). Efficacy and safety of electroacupuncture on treating depression-related insomnia: A study protocol for a multicentre randomised controlled trial. BMJ Open, 9(4).

Yin, X., Xu, J., Dong, B., Ma, J., Chen, Z., Yin, P., Wu, J., et al. (2016). Efficacy and Safety of Electroacupuncture on Treating Depression Related Sleep Disorders: Study Protocol of a Randomized Controlled Trial. Evidence-based Complementary and Alternative Medicine, 2016.

Zhao, F. Y., Fu, Q. Q., Spencer, S. J., Kennedy, G. A., Conduit, R., Zhang, W. J., & Zheng, Z. (2021). Acupuncture: A promising approach for comorbid depression and insomnia in perimenopause. Nature and Science of Sleep, 13(October), 1823–1863.

Zhao, J., Liu, H., Wu, Zhiguo, Wang, Yun, Cao, T., Lyu, D., Huang, Q., et al. (2021). Clinical features of the patients with major depressive disorder co-occurring insomnia and hypersomnia symptoms: a report of NSSD study. Sleep Medicine. Elsevier Ltd, 81, 375–381.

Zhao, K. (2013). Acupuncture for the treatment of insomnia. *International review of neurobiology*, *111*, 217-234.

Zheng, Y., He, J., Guo, L., Yao, L., Zheng, X., Yang, Z., Xia, Y., et al. (2019). Transcriptome analysis on maternal separation rats with depression-related manifestations ameliorated by electroacupuncture. Frontiers in Neuroscience, 13(APR), 1–13.

Zhou, X.-F., Li, Y., Zhu, H., & Chen, L.-L. (2013). Impacts of acupuncture at twelve meridians acupoints on brain waves of patients with general anxiety disorder. Zhongguo Zhen Jiu, 33(5), 395–398.

Zhou, Y.-L., Gao, X.-Y., Wang, P.-Y., & Ren, S. (2012). Effect of acupuncture at different acupoints on expression of hypothalamic GABA and GABA(A) receptor proteins in insomnia rats. Zhen Ci Yan Jiu, 37(4), 302–307.

Zung, W. W. (1971). A rating instrument for anxiety disorders. Psychosomatics: Journal of Consultation and Liaison Psychiatry.

Zuppa, C., do Prado, C. H., Wieck, A., Zaparte, A., Barbosa, A., & Bauer, M. E. (2015). Acupuncture for sleep quality, BDNF levels and immunosenescence: a randomized controlled study. Neuroscience Letters, 587, 35-40.

JAWATANKUASA ETIKA PENYELIDIKAN (MANUSIA) – JEPeM USM
UNIVERSITI SAINS MALAYSIA

RESEARCH INFORMATION (TRANSLATED ENGLISH COPY)

Research Title: Efficacy and Safety of Low-dose Laser Acupuncture on Treating Insomnia Associated with Depression: A Randomised Controlled Trial

Name of main and co-Researcher: Dr. Mohammad Farris Iman Leong Bin Abdullah, (MMC: 43103), Qiu Qiyue

INTRODUCTION

We extend an invitation for you to volunteer in a research study aimed at assessing the effectiveness and safety of laser acupuncture in treating insomnia symptoms that occur alongside depression. Participants will be required to complete a series of questionnaires, including the Pittsburgh Sleep Quality Index (PSQI), the Hamilton Depression Rating Scale (HAMD), the Insomnia Severity Index (ISI), and the Self-Assessment Anxiety Scale (SAS). Additionally, your sleep patterns will be tracked using an activity logger, and blood tests will be conducted to measure the levels of 5-hydroxytryptophan (5-HT) and corticosterone (CORT). Upon entering the study, you will be administered standard antidepressant therapy, with careful documentation of its usage.

Before consenting to join the study, please ensure you have thoroughly read and understood all pertinent information provided. Upon agreeing to participate, a copy of this consent form will be given to you for your personal records.

The duration of your participation in this research is anticipated to be around 18 weeks, with the study aiming to enroll a total of up to 90 participants.

PURPOSE OF THE STUDY

The study's primary purpose is to evaluate the effectiveness and safety of low-dose laser acupuncture (LLA) in alleviating insomnia symptoms among patients with depressive disorders comprehensively. This involves a systematic assessment of LLA's impact on sleep quality and mood disorders, employing tools such as the Pittsburgh Sleep Quality Index (PSQI),

Self-Rating Anxiety Scale (SAS), Hamilton Depression Rating Scale (HAMD), alongside actigraphy for a detailed analysis of sleep patterns, including efficiency, awakening frequency, and total duration, paired with evaluations using the Insomnia Severity Index (ISI). Additionally, the study aims to explore changes in serum levels of Corticosterone (CORT) and 5-Hydroxytryptamine (5-HT) pre, during, and post-treatment, compare the effectiveness of various treatment combinations of LLA with antidepressant drugs, and assess the post-treatment safety profile of laser acupuncture.

PARTICIPANTS CRITERIA

The research team members will discussed your eligibility to participate in this study. It is important that you are completely truthful with the staff including your health history.

This study will enroll participants who meet the following criteria:

- Hospitalized patients diagnosed with major depressive disorder;
- Individuals of any gender, aged between 18 and 70 years;
- Those reporting insomnia during the initial screening;
- A Pittsburgh Sleep Quality Index (PSQI) score greater than 7;
- A 17-item Hamilton Depression Rating Scale (HAMD) score ranging from 20 to 35;
- No consumption of hypnotic drugs or receipt of acupuncture treatment in the past month;
- Absence of cognitive or communication impairments;
- Agreement to participate in random group allocation and to provide signed informed consent.

The study will exclude individuals characterized by:

- A pronounced risk of suicide, as determined by a specialist;
- A history of schizophrenia, bipolar disorder, or other psychiatric conditions;
- Serious alcohol or substance abuse problems;
- Significant liver or kidney dysfunction, uncontrollable tumors, or major cerebrovascular diseases;
- Current pregnancy or breastfeeding status;
- Potential for poor adherence to the study protocol or an aversion to laser acupuncture treatments.

STUDY PROCEDURES

The study involving all enrolled participants mainly includes two aspects. The first is the process related to medical treatment. Initially, participants will receive conventional inpatient treatment, primarily involving the prescription of Selective Serotonin Reuptake Inhibitors (SSRIs), among others.

Second, participants will undergo a comprehensive assessment involving a variety of medical and psychological evaluations. These evaluations include the Pittsburgh Sleep Quality Index (PSQI) for assessing sleep quality, the Self-Rating Anxiety Scale (SAS) for anxiety, the

Hamilton Depression Rating Scale (HAMD) for depressive symptoms, the Insomnia Severity Index (ISI) for the severity of insomnia, and actigraphy data analysis to quantitatively evaluate sleep patterns. Additionally, blood samples will be collected to measure levels of serum corticosterone (CORT) and 5-hydroxytryptamine (5-HT) as part of the baseline assessment. The entire process is designed to take approximately 30 minutes.

Third, articipants will be allocated randomly into one of three groups: the group undergoing low-dose laser acupuncture (LLA), the sham laser acupuncture group (SLA), and the standard treatment control group. Following this allocation, participants will undergo subsequent evaluations including the Pittsburgh Sleep Quality Index (PSQI), the Hamilton Depression Rating Scale (HAMD), the Self-Rating Anxiety Scale (SAS), the Insomnia Severity Index (ISI), actigraphy data analysis, and measurements of Serum 5-HT and Serum CORT. These assessments will be conducted at 4 weeks, 6 weeks, and 18 weeks following the initial baseline evaluation.

Below are descriptive of the interventions that will be provided according to the groups you assigned to:

(1) Low-dose Laser Acupuncture (LLA): This innovative treatment method combines traditional acupuncture techniques with modern laser technology, by delivering a gentle laser beam to specific acupuncture points, it enhances the body's self-healing capabilities for symptom relief and disease treatment. It is particularly suited for patients who are apprehensive about traditional needle acupuncture or those seeking a non-invasive treatment approach. Low-dose laser is renowned for its properties in promoting tissue regeneration, reducing inflammation, and alleviating pain. Therapeutic points in the LLA group included Baihui (GV20), Yintang (GV29), Anmian (EX-HN22), Shenmen (HT7), Neiguan (PC6), Sanyinjiao (SP6), and Taichong (LR3). To avoid interference, they will be treated in a secluded environment. Additionally, they are required to wear eye masks before and during the trial for safety. After sterilizing these points, a probe will be secured with medical tape, and the device used will be the xS-998D06 semiconductor laser acupoint therapy device produced by Nanjing Xiaosong Medical Instruments. The treatment mode is set to 780 nm and 5 mW output, conducted over six consecutive weeks, with five days of treatment per week, one session per day, each lasting 20 minutes, with a two-day rest interval between treatment weeks.

(2) Sham Laser Acupuncture (SLA): SLA is designed to replicate the treatment process of LLA without emitting a laser beam, employing the same treatment procedures to ensure consistency in participant experience. Patients will also be treated in a secluded environment. Furthermore, they are required to wear eye masks before and during the trial for safety. Before each treatment session, the acupoints will be sterilized, and the treatment device's probe will be secured to the acupoints with medical tape but will remain non-emissive throughout the 20-minute treatment, ensuring no laser output. This method will be carried out for six consecutive weeks, with five days of treatment per week, one session per day, each lasting 20 minutes, with a two-day rest interval between treatment weeks. This approach helps in evaluating the efficacy of LLA by distinguishing between real treatment effects and placebo

effects, a critical step in accurately assessing the clinical value of the method and promoting advancements in medical treatments.

(3) Control Group: A medication group will serve as the control in this study, established to identify any significant differences in treatment outcomes between the LLA group and a conventional control group. This setup aims to provide clearer insights into the potential benefits of LLA for managing conditions such as insomnia and depressive disorders. Over an initial period up to 6 weeks, participants in the control group will be prescribed Selective Serotonin Reuptake Inhibitors (SSRIs), allowing for a comparative analysis between LLA , s therapeutic efficacy and that of standard pharmacological treatments for these conditions.

RISKS

Participation in this study, aimed at treating insomnia related to depression through low-dose laser acupuncture, carries minimal risk. However, should you feel emotionally distressed after participating in any part of the study, we will offer a referral to a counselor at the 2nd Affiliated Hospital of Xinxiang Medical University, Henan, China. Moreover, if symptoms of depression and anxiety persist after the study's completion, a referral will be made to the Psychiatry Department of the same institution for additional evaluation and necessary treatment. Please communicate any concerns or significant information that might affect your participation in the study to the research team.

Before any referrals, emergency medication may be provided to alleviate acute distress. Additionally, participants experiencing mental health disturbances during the study may receive counseling services from the Henan Mental Hospital, Henan, China. The confidentiality of all personal information will be strictly maintained, and participants are assured that they will receive all entitled benefits even if they choose to withdraw from the study. Upon the study's conclusion, participants may also be guided towards supportive communities, including specialized support groups for individuals dealing with insomnia and depression, to facilitate ongoing recovery and support.

Specific risk may also occur in different groups of insomnia associated with depression subjects:

(a) For Unemployed Participants: Those dealing with unemployment may face financial hardships and barriers to finding employment. We plan to refer such participants to social workers for financial support and connect them with job managers and occupational therapists within the community psychiatry team at the Department of Psychiatry, 2nd Affiliated Hospital, XXMU. These professionals are equipped to assist in locating appropriate job opportunities and providing job training to enhance participants' employability.

(b) For Participants with Families: Individuals with families may inadvertently subject their loved ones to a range of psychosocial challenges. To address this, we will offer support to the family members through mental health screenings and direct them to counselors or the

Department of Psychiatry at the 2nd Affiliated Hospital, XXMU, for further management. This measure aims to protect the well-being of all family members involved.

(c) For Those Experiencing Marital Discord: Participants facing marital challenges will be encouraged to seek marital counseling services provided by the Department of Psychiatry at the 2nd Affiliated Hospital, XXMU. Such counseling is designed to resolve underlying issues, thereby promoting healthier and more supportive relationship dynamics.

(d) For Placebo Group Participants: Those assigned to the placebo group might experience mental health disturbances throughout the study. For participants who decide to withdraw from the study due to any psychological discomfort, we will offer immediate referral to the Department of Psychiatry at the 2nd Affiliated Hospital, XXMU, for further evaluation and the necessary treatment.

REPORTING HEALTH EXPERIENCES.

Please contact, at any time, the following researcher if you experience any health problem either directly or indirectly related to this study.

Dr. Mohammad Farris Iman Leong Bin Abdullah **[MMC Registration No._43103]** at +604-5622482 or +6018-6669950.

PARTICIPATION IN THE STUDY

Your taking part in this study is entirely voluntary. You may refuse to take part in the study or you may stop your participation in the study at anytime, without any penalty or loss of benefits to which you are otherwise entitled. Your participation also may be stopped by the research team without your consent if in any form you have violated the study eligibility criteria. The research team member will discussed with you if the matter arises.

POSSIBLE BENEFITS [Benefits to Individual, Community, and University]

Participation in this research is offered at no cost to you. Engaging in this study on laser acupuncture treatment for insomnia associated with depression offers several direct benefits, including: (a) Gaining valuable insights into your mental health status, particularly concerning insomnia related to depression. These insights are vital for your mental health care. (b) The opportunity to receive laser acupuncture treatment, which is anticipated to improve your sleep quality and reduce symptoms of depression. This could indirectly enhance your daily functioning, increase the likelihood of achieving stable employment, and foster improved relationships with family members by addressing the root causes of your insomnia.

The outcomes of this research are expected to benefit the community by demonstrating the effectiveness of laser acupuncture as a non-pharmacological intervention for managing

insomnia associated with depression. This could lead to recommendations for incorporating laser acupuncture into existing treatment plans for individuals suffering from this condition, filling a significant gap in the current treatment landscape for insomnia associated with depression.

No financial compensation will be provided for participating in this study. While there is no insurance coverage specific to participation, the research team will fully cover any costs related to the treatment and rehabilitation of injuries or conditions that occur as a direct result of participation in the study. Participants may be eligible for reimbursement for travel expenses incurred during the study period. This research does not aim to produce any commercial products; rather, it focuses on enhancing the understanding and treatment of insomnia associated with depression.

QUESTIONS

If you have any question about this study or your rights, please contact;

Dr. Mohammad Farris Iman Leong Bin Abdullah
Primary investigator
Department of Community Health
Advanced Medical and Dental Institute
Universiti Sains Malaysia
SAINS@BERTAM
13200 Kepala Batas
Pulau Pinang
Malaysia
+60186669950

Qiu Qiyue
Co-primary investigator
Department of Traditional Medicine and Herbal Medicine
School of Pharmaceutical Sciences
Universiti Sains Malaysia
11700 Gelugor
Pulau Pinang
Malaysia
+60175753577

If you have any questions regarding the Ethical Approval or any issue / problem related to this study, please contact;

Mr. Mohd Bazlan Hafidz Mukrim
Secretary of Human Research Ethics Committee USM
Division of Research & Innovation (R&I)
USM Health Campus
Tel. No. : +609-767 2354 / +609-767 2362
Email : bazlan@usm.my

OR

Miss Nor Amira Khurshid Ahmed
Secretariat of Human Research Ethics Committee USM
Research Creativity & Management Office (RCMO)
USM Main Campus, Penang
Tel. No. : +604-6536537
Email : noramira@usm.my

The investigators serve only as investigator of this study and they are not the one who provide service to the participants.

CONFIDENTIALITY

Your information will be kept confidential by the researchers and will not be made publicly available unless disclosure is required by law.

Data obtained from this study that does not identify you individually will be published for knowledge purposes.

Your original records may be reviewed by the researcher, the Ethical Review Board for this study, and regulatory authorities for the purpose of verifying the study procedures and/or data. Your information may be held and processed on a computer. Only research team members are authorized to access your information. The information will be stored for 2 years after completion of the study before it is discarded following standard procedures. While any biological sample (blood and feces) will be discarded following standard procedures once analysis is completed in the study. Any future possible use of the data and specimen collected for research purpose will be communicated to you by the research team and you may refuse to consent for future use and we will discard the data and specimen accrodingly.

The feedback on the study findings will be informed by the research team after completion of the study upon request from the participants.

By signing this consent form, you authorize the record review, information storage and data process described above.

SIGNATURES

To be entered into the study, you or a legal representative must sign and data the signature page **[ATTACHMENT S or or ATTACHMENT P]**

ATTACHMENT S

Subject Information and Consent Form
(Signature Page)

Research Title: Efficacy and Safety of Low-dose Laser Acupuncture on Treating Insomnia Associated with Depression: A Randomised Controlled Trial

Name of main and co-Researcher: Dr. Mohammad Farris Iman Leong Bin Abdullah (MMC: 43103), Qiu Qiyue

To become a part this study, you or your legal representative must sign this page. By signing this page, I am confirming the following:

- I have read all of the information in this Patient Information and Consent Form **including any information regarding the risk in this study** and I have had time to think about it.
- All of my questions have been answered to my satisfaction.
- I voluntarily agree to be part of this research study, to follow the study procedures, and to provide necessary information to the doctor, nurses, or other staff members, as requested.
- I may freely choose to stop being a part of this study at anytime.
- I have received a copy of this Participant Information and Consent Form to keep for myself.

____

Participant Name

____

Participant I.C No

____ ________________________

Signature of Participant or Legal Representative **Date** (dd/MM/yy)

____

Name of Individual
Conducting Consent Discussion

Signature of Individual Date (dd/MM/yy)
Conducting Consent Discussion

Name & Signature of Witness Date (dd/MM/yy)

Note: i) All participants who are involved in this study will not be covered by insurance.

ATTACHMENT P

Participant's Material Publication Consent Form
Signature Page

Research Title: Efficacy and Safety of Low-dose Laser Acupuncture on Treating Insomnia Associated with Depression: A Randomised Controlled Trial

Name of main and co-Researcher: Dr. Mohammad Farris Iman Leong Bin Abdullah (MMC: 43103), Qiu Qiyue

To become a part this study, you or your legal representative must sign this page.

By signing this page, I am confirming the following:

- I understood that my name will not appear on the materials published and there have been efforts to make sure that the privacy of my name is kept confidential although the confidentiality is not completely guaranteed due to unexpected circumstances.

- I have read the materials or general description of what the material contains and reviewed all photographs and figures in which I am included that could be published.

- I have been offered the opportunity to read the manuscript and to see all materials in which I am included, but have waived my right to do so.

- All the published materials will be shared among the medical practitioners, scientists and journalist world wide.

- The materials will also be used in local publications, book publications and accessed by many local and international doctors world wide.

- I hereby agree and allow the materials to be used in other publications required by other publishers with these conditions:

- The materials will not be used as advertisement purposes nor as packaging materials.

- The materials will not be used out of contex – i.e.: Sample pictures will not be used in an article which is unrelated subject to the picture.

____

Participant Name

__________________	____________________	____________________
**Participant I.C No.**	**Participant's Signature**	**Date** (dd/MM/yy)

____	____________________
**Name and Signature of Individual**<br>Conducting Consent Discussion	**Date** (dd/MM/yy)

Note: i) All participants who are involved in this study will not be covered by insurance.

研究伦理委员会（人类） – JEPeM USM

马来西亚理科大学

研究信息（中文译本）

研究标题： 低剂量激光针灸治疗抑郁症相关失眠的疗效与安全性： 一项随机对照试验

主要研究人员及合作研究人员： 穆罕默德 ·法里斯 ·伊曼 ·梁 ·阿 卜杜拉 博士（MMC: 43103）, 邱琪越

介绍

我们邀请您自愿参与本项干预性研究。旨在评估激光针灸治疗与抑郁症相关的失眠的有效性和安全性。 参与本研究， 您将需要完成一系列问卷， 包括匹兹堡睡眠质量指数（PSQI）、 汉密尔顿抑郁症评分量表（HAMD）、 失眠严重指数（ISI） 和自评焦虑量表（SAS）， 同时通过活动记录仪监测您的睡眠情况， 并检测血清中 5-羟色胺（5-HT） 和皮质酮（CORT） 的水平。 在入院时， 您将接受常规的抗抑郁药物治疗， 并对药物使用情况进行详细记录。

在同意参加研究之前，请确保您已经阅读并理解了有关本研究的所有信息。如果您同意参与，您将收到一份本同意书的副本， 以备您的记录。

您参与本研究的预期时间大约为 18 周， 本研究计划招募多达 90 名参与者。

研究目的

本研究的主要目的是全面评估低剂量激光针灸（LLA）缓解抑郁症患者失眠症状的有效性和安全性。 这包括系统地评估 LLA 对睡眠质量和情绪障碍的影响， 采用匹兹堡睡眠质量指数（PSQI）、自评焦虑量表（SAS）、 汉密尔顿抑郁量表（HAMD） 等工具， 以及通过活动监测对睡眠模式进行详细分析， 包括睡眠效率、 觉醒频率和总睡眠时长， 并结合使用失眠严重指数（ISI） 进行评估。 此外， 研究旨在探索治疗前、 中、 后血清皮质酮（CORT） 和 5-羟色胺（5-HT） 水平的变化，比较 LLA 与抗抑郁药物不同治疗组合的有效性， 以及评估激光针灸治疗后的安全性。

受试者标准

研究团队成员将讨论您参与本研究的资格。请您务必对工作人员完全诚实，包括您的健康史。本研究将纳入符合以下标准的受试者：

- 诊断为重度抑郁障碍的住院病人。
- 年龄介于 18 至 70 岁之间， 不限性别。
- 初筛时报告存在失眠症状者。
- 匹兹堡睡眠质量指数（PSQI） 评分超过 7 分。
- 17 项汉密尔顿抑郁评分量表（HAMD） 得分在 20 至 35 分之间。

- 过去一个月内没有使用过催眠药物或接受过针灸治疗。
- 没有认知或沟通上的障碍。
- 同意按随机分组原则参与研究并签署知情同意书。

本研究将排除符合以下标准的受试者：

- 由专家评估确定存在显著自杀风险者。
- 有精神分裂症、 躁郁症或其他精神疾病史的个体。
- 存在严重的酒精或药物滥用问题。
- 有重大的肝脏或肾脏功能障碍、 不可控制的肿瘤或重要脑血管疾病者。
- 正在怀孕或哺乳期的个体。
- 对研究方案依从性低或对激光针灸治疗持有抵触情绪的人。

研究流程

研究程序

所有入组受试者的研究开展主要包含两方面。 第一是药物治疗相关过程。 首先， 受试者将接受常规住院治疗， 主要包括处方选择性血清素再摄取抑制剂等。

其次，参与者将接受一系列包括医学和心理评估在内的综合评估。这些评估包括利用匹兹堡睡眠质量指数（PSQI）来评估睡眠质量、 自评焦虑量表（SAS）评估焦虑情况、 汉密尔顿抑郁评分量表（HAMD）评估抑郁症状、 失眠严重度指数（ISI）评估失眠的严重程度， 以及通过活动量表数据分析定量评估睡眠模式。 此外， 还将采集血样， 作为基线评估的一部分， 测量血清皮质醇（CORT） 和 5-羟色胺（5-HT） 的水平。 整个过程预计将耗时约 30 分钟。

第三， 参与者将被随机分配到三个组之一：低剂量激光针灸（LLA）组、假激光针灸（SLA）组和标准治疗对照组。 分组后， 参与者将接受随后的评估， 包括匹兹堡睡眠质量指数（PSQI）、 汉密尔顿抑郁评分量表（HAMD）、 自评焦虑量表（SAS）、 失眠严重度指数（ISI）、 活动量表数据分析， 以及血清 5-HT 和血清 CORT 的测量。 这些评估将在初始基线评估后的第 4 周、 第 6 周和第 18 周进行。

以下是本研究的干预性措施（根据您被分到的研究小组所提供）的描述性说明：

（1） 低剂量激光针灸（LLA）：这种创新治疗方法将传统的针灸疗法与现代激光技术相结合，通过向特定的针灸点输送温和的激光光束，增强机体的自我恢复能力，用于缓解症状和治疗疾病。特别适合对传统针灸持有顾虑或寻求非侵入式治疗方案的患者，低剂量激光以促进人体组织再生、 减少炎症和缓解疼痛的特性而闻名。 LLA 组治疗穴位包括百会穴（GV20）、 印堂穴（GV29）、 安眠穴（EX-HN22）、神门穴（HT7）、 内关穴（PC6）、三阴交穴（SP6）和太冲穴（LR3）。为避免交流他们将在一个封闭的环境中接受治疗。 此外， 他们还被要求在试验前和试验期间戴上眼罩。 在对这些穴位进行消毒，将使用医用胶带固定探头，仪器为南京小松医疗器械公司生产的 xS-998D06 半导体激光穴位治疗仪。 治疗模式设定为 780nm 和 5mW 输出， 连续六周， 每周进行 5 天治疗， 每天治疗一次， 每次治疗持续 20 分钟， 治疗间隔休息两天。

(2) 假激光针灸（SLA）：旨在复制 LLA 的治疗过程而不发射激光光束， 采用相同的治疗程序以确保参与者的体验一致性。 患者同样将处于一个封闭的环境中接受治疗。 此外， 他们还被要求在试验前和试验期间戴上眼罩。 在每次治疗会议之前， 将对穴位进行消毒， 治疗仪的探头用医用胶带固定在穴位上， 但在 20 分钟的治疗中保持不发射状态， 确保没有激光输出。 这种方式连续进行六周， 每周进行 5 天， 每天一次， 每次持续 20 分钟， 治疗间隔休息两天。 有助于通过区分真正的治疗效果和安慰剂效应来评估 LLA 的有效性， 这是准确评估该方法临床价值并促进医疗治疗进步的关键步骤。

(3) 对照组： 本研究将设立一个药物组作为对照组， 以识别LLA 组与传统对照组之间的治疗结果是否有显著差异。 此设置旨在为 LLA 管理如失眠和抑郁症提供更清晰的见解。 在住院的6 周期间， 对照组的参与者将被处方选择性血清素再摄取抑制剂 （ SSRIs ）， 允许对 LLA 的治疗效果与这些标准药物治疗进行比较分析。

风险

参与本项研究的风险很小。但是如果您在回答问卷后出现情绪障碍，我们建议您向中国河南新乡医学院第二附属医院的咨询师进行咨询。如果您在研究结束后仍出现抑郁和焦虑症状， 我们建议您到新乡医学院第二附属医院的精神科接受进一步评估及治疗。如果您遇到任何问题或获得任何会改变您参与研究的重要信息， 请告知本研究团队。

参与本研究， 旨在通过低剂量激光针灸治疗与抑郁症相关的失眠， 风险极小。 然而， 如果您在参与研究的任何部分后感到情绪困扰，我们将提供转介至中国河南省新乡医学院第二附属医院的咨询师。 此外， 如果在研究完成后抑郁和焦虑症状仍然持续， 将会被转介到同一机构的精神科进行额外评估和必要的治疗。 请将任何可能影响您参与研究的关注点或重要信息通知研究团队。

在进行任何转介之前， 可能会提供急救药物， 以缓解急性困扰。 此外， 参与研究期间经历心理健康干扰的参与者，可以从中国河南省河南精神病医院接受咨询服务。所有个人信息的保密性将得到严格维护， 且参与者即便选择退出研究， 也确保能够获得所有应得的利益。 研究结束时，参与者还可能被引导加入支持性社群，包括为那些处理失眠和抑郁问题的人设立的专业支持小组，以促进持续的康复和支持。

具体的风险也可能发生在不同的抑郁症伴失眠患者群体中：

(a) 失业参与者： 我们计划为其提供财务支持， 并通过新乡医学院第二附属医院精神科的社区团队帮助寻找工作及提供职业培训。

(b) 有家庭的参与者： 我们将为家庭成员提供心理健康筛查和咨询支持， 以保护家庭成员的福祉。

(c) 经历婚姻问题的参与者： 我们鼓励其寻求新乡医学院第二附属医院精神科的婚姻咨询服务， 促进健康的关系动态。

(d) 安慰剂组参与者： 对因心理不适想退出研究的参与者， 我们将立即转介至新乡医学院第二附属医院精神科进行评估和治疗。

健康问题报告

如果您遇到任何与本研究直接或间接相关的健康问题， 请随时联系以下研究人员：

穆罕默德·法里斯·伊曼·梁·阿 卜杜拉 （ 博士 ） **[MMC 编号 <u>43103</u>]** 电话： +604-5622482 / +6018-6669950。

` **参与研究**

您的参与是完全自愿的。您可以在任何时候拒绝参加本研究或停止参加本次研究，您本应享有的利益不会受到任何处罚或损失。如果您以任何形式违反了研究资格标准，研究团队也可能会在未经您同意的情况下停止您的参与。 如果出现问题， 研究团队成员将与您讨论。

潜在获益 [个人、 社会、 大学的受益]

参与本项研究无需您承担任何费用。加入这一针对与抑郁相关失眠的激光针灸治疗研究，将为您带来诸多直接益处， 具体包括： (a) 获得关于您心理健康状态的宝贵洞见， 尤其是针对与抑郁相关的失眠问题， 这对于您的心理健康护理极为重要； (b) 机会接受激光针灸治疗， 预计此治疗将改善您的睡眠质量并缓解抑郁症状，从而可能间接提升您的日常功能表现，增加获得稳定工作的可能性， 并通过解决失眠的根源， 改善与家人的关系。

本研究预期将通过证明激光针灸作为非药物介入手段管理与抑郁相关失眠的有效性，为社区带来益处。这可能促成将激光针灸纳入现有治疗方案的建议，填补当前治疗与抑郁相关失眠的重大空白。

参与本研究将不提供任何财务补偿。尽管没有特别针对参与者的保险覆盖，但研究团队将完全负担由参与研究直接引起的任何治疗和康复的费用。参与者在研究期间发生的旅行费用可能会得到报销。 本研究旨在深化对与抑郁相关失眠的理解和治疗方法， 不打算开发任何商业产品。

答疑

如果您对本研究或您的权利有任何疑问， 请联系我们：

穆罕默德·法里斯·伊曼·梁·阿 卜杜拉 博士
主要研究人员
公共卫生系
高级医学和牙科研究所
马来西亚理科大学
SAINS@BERTAM
13200 加巴拉巴打
槟城
马来西亚
+60 18-6669950

邱琪越
共同主要研究人员
传统医学与草药学系
药学院
马来西亚理科大学
11700 牛汝莪
槟城
马来西亚
+60 175753577

如果您对伦理审批或与本研究相关的任何问题有任何疑问， 请联系：

穆罕默德·巴兹兰·哈菲兹·穆克里姆 先生
马来西亚理科大学人类研究伦理委员会 秘书
研究与创新部（R&I）
马来西亚理科大学健康学院
电话号码： +609-767 2354 / +609-767 2362
邮箱： bazlan@usm.my

或者

诺尔·阿米拉·胡尔希德·艾哈迈德 女士
马来西亚理科大学人类研究伦理委员会秘书处
研究创新与管理办公室（RCMO）
马来西亚理科大学主校区， 槟城
电话号码： +604-6536537
邮箱： noramira@usm.my

研究人员只是本项目的研究开展者， 并不是为受试者提供服务的人。

保密性

研究人员将对您的信息保密， 除非法律要求披露， 否则不会公开。

从本研究中获得的数据不会披露您的个人身份， 数据将出于知识目的发表。

您的原始记录可能会被研究人员、本研究的伦理审查委员会和监管机构审查， 以核实研究程序和/或数据。 您的信息可能会在计算机上储存和处理。 只有研究团队成员有权查阅您的信息 。 这些信息将在研究完成后存储两年， 然后按照标准程序弃置。 而任何生物样本(血液和粪便)将在研究分析完成后按照标准程序弃置。本研究团队将会告知您相关资料及样本出于研究目的的日后使用情况， 您可以拒绝日后使用， 我们亦会相应弃置该资料及样本。

在研究结束后， 应受试者的要求， 研究团队将告知研究结果的反馈。

签署本知情同意书， 即表示您已授权上述的记录审查、 信息存储和数据处理。

签字

同意参与本研究， 您或法定代理人必须在签名页上签字并填写资料。

[附件 S 或 附件 P]

附件 S

受试者信息及知情同意书
（签字页）

研究标题：　　低剂量激光针灸治疗抑郁症相关失眠的疗效与安全性：一项随机对照试验

主要研究人员及合作研究人员：穆罕默德·法里斯·伊曼·梁·阿 卜杜拉 博士（MMC:43103），邱琪越

加入本研究前，您或您的法定代理人须在本页签名。通过签署本页，本人确认以下内容：

- 我已经阅读了这份受试者信息及知情同意书中的全部内容，**包括关于本研究风险的所有信息**，并且我已在足够的时间内对以上内容进行思考。
- 我对于本研究相关的所有问题都得到了满意回答。
- 我自愿同意参与本研究，遵循研究程序，并根据要求向医生、护士或其他工作人员提供必要的信息。
- 我可以随时自由选择停止参与这项研究。
- 我已收到受试者信息及知情同意书的副本，并由我本人保存。

____

受试者姓名

____

受试者编号

______________________________　　　　______________

受试者（或法定代理人）**签字**　　　　**日期**（年/月/日）

____

个人姓名
进行同意讨论

______________________________　　　　______________

个人签字　　　　**日期**（年/月/日）
进行同意讨论

______________________________　　　　______________

见证人姓名及签字　　　　**日期**（年/月/日）

备注：　i)　所有参与本研究的受试者将不在保险范围内。

附件 P

受试者资料发表同意书
签字页

研究标题：　低剂量激光针灸治疗抑郁症相关失眠的疗效与安全性：一项随机对照试验

主要研究人员及合作研究人员：穆罕默德·法里斯·伊曼·梁·阿卜杜拉 博士（MMC:43103）、邱琪越

加入本研究前，您或您的法定代理人须在本页签名。通过签署本页，本人确认以下内容：

- 我已知晓本人的姓名将不会出现在出版的资料上。同时，即使由于不可预估情况而无法完全保障保密性，研究人员已经努力确保本人姓名的隐私保密。
- 我已经阅读了资料或资料所包含的一般描述，并查阅了所有包括我在内的可能被发表的照片和图像。
- 我已经被提供机会阅读论文手稿，并查看所有包含我的资料，但我放弃了这样做的权利。
- 本研究所有出版的资料将在全世界的医疗从业者、科学家和记者之间共享。
- 本研究的资料也将被用于国内外医学杂志出版物及图书出版物，并且供全世界范围内的医生查阅。
- 我同意并允许在符合以下条件的其他出版商的出版物中使用本研究的资料：
- 研究资料的内容将不会被用于广告目的或产品包装。
- 不得断章取义地使用研究资料——例如：样本图片不得被用于与图片无关的文章中。

____

受试者姓名

______________　______________　______________

受试者编号　**受试者签字**　**日期**（年/月/日）

______________________________　______________

个人姓名及签字　**日期**（年/月/日）

进行同意讨论

备注：　i)　所有参与本研究的受试者将不在保险

Pittsburgh Sleep Quality Index (PSQI)					
**Note:** The following questions only relate to your sleep habits in the past month. You should give accurate answers about how much sleep you have had on most days and nights during the past month. You should answer all questions. Please choose the one that best suits your situation and outline √ . Thank you for your participation!					
Symptoms		Score			
		0	1	2	3
1. Nearly 1 month, go to bed at night usually at (　　) o 'clock					
2. In nearly one month, it usually takes (　　) minutes from going to bed to fall asleep		≤15min	16~30min	31~60min	≥60min
3. In the past month, I usually get up at (　　) o 'clock in the morning					
4. In recent 1 month, usually actual sleep (　　) h per night (not equal to bed time)					
5、	a. Difficulty falling asleep (unable to fall asleep within 30 minutes)	No	<1x/wk	1-2x/wk	≥3x/wk
	b. Easy to wake up at night or early	No	<1x/wk	1-2x/wk	≥3x/wk
	c. Go to the bathroom at night	No	<1x/wk	1-2x/wk	≥3x/wk
	d. Poor breathing	No	<1x/wk	1-2x/wk	≥3x/wk
	e. Coughing or snoring loudly	No	<1x/wk	1-2x/wk	≥3x/wk
	f. Feeling cold	No	<1x/wk	1-2x/wk	≥3x/wk
	g. Feel hot	No	<1x/wk	1-2x/wk	≥3x/wk
	h. Nightmares	No	<1x/wk	1-2x/wk	≥3x/wk
	i. Pain and discomfort	No	<1x/wk	1-2x/wk	≥3x/wk
	j. Other things that affect sleep	No	<1x/wk	1-2x/wk	≥3x/wk
6. In the past month, generally speaking, do you think your sleep quality is:		Very good	Good	Fair	Poor
7. In the past month, your use of drugs for hypnosis:		No	<1x/wk	1-2x/wk	≥3x/wk
8. In the past month, do you often feel sleepy?		No	<1x/wk	1-2x/wk	≥3x/wk
9. Do you have insufficient energy to do things in the past month?		None	Occasionally	Sometimes	Often
Evaluator:			Total Score:		

Hamilton Depression Scale (HAMD)					
In the past week, have you experienced the following symptoms listed in the questionnaire? Please rate the extent to which each symptom has affected you, selecting one level from "0" indicating no impact at all, to "4" indicating extreme impact. The closer to 0, the less affected you are, while the closer to 4, the more affected you are. Please select the most appropriate level from "0 to 4" and mark it with a checkmark (√). Thank you for your participation!					
Symptoms	Degree of Symptoms				
	0	1	2	3	4
1. Depressed mood (feeling sad, hopeless, helpless, worthless)					
2. Guilt feelings					
3. Suicidal thoughts					
4. Difficulty falling asleep					
5. Light sleep					
6. Early morning awakening					
7. Work and interests					
8. Slowness					
9. Agitation					
10. Psychic anxiety					
11. Somatic anxiety					
12. Gastrointestinal symptoms					
13. General somatic symptoms					
14. Sexual symptoms (such as loss of libido)					
15. Hypochondriasis					
16. Weight loss					
17. Insight					
18. Diurnal variation					
19. Dissociative or reality dissociation					
20. Paranoid symptoms					
21. Obsessive thoughts and compulsive behaviors					
22. Feeling of diminished ability					
23. Feelings of despair					
24. Feelings of inferiority					
Evaluator:	Total score:				

Self-Assessment Scale for Anxiety (SAS)				
Please carefully read each item, understand its meaning, and then, based on your actual feelings over the past week, primarily assess the frequency of occurrence of the symptoms. The assessment criteria are: none or rarely; sometimes; most of the time; almost always. Please mark a check mark (√) in the position that best fits your situation. Thank you for your participation!				
Symptoms	Degree of Symptoms			
	Rarely	Some	Most of	Almost
1. I feel more nervous than usual.				
2. I feel scared for no reason.				
3. I am easily upset or feel panicky.				
4. I feel like something bad is going to happen.				
5. I feel that everything is fine and nothing bad				
6. My hands and feet tremble uncontrollably.				
7. I am troubled by headaches, neck pain, and				
8. I feel weak and tired easily.				
9. I feel calm and can sit quietly easily.				
10. I feel my heart beating very fast.				
11. I am troubled by bouts of dizziness.				
12. I have fainting spells or feel like I might faint.				
13. Breathing in and out feels very easy.				
14. My hands and feet are numb and tingling.				
15. I am troubled by stomach pain and				
16. I need to urinate frequently.				
17. My hands are often dry and warm.				
18. My face feels hot and flushed.				
19. I fall asleep easily and sleep well all night.				
20. I have nightmares.				
Evaluator:	Total score:			

Insomnia Severity Index (ISI)					
Please read each item carefully and fully understand its meaning. Then, answer the following questions based on the past month. Select the option that most accurately describes your sleep condition and mark it with a check (√). Thank you very much for your participation and cooperation!					
Symptoms	Degree of symptoms				
	0	1	2	3	4
1. Difficulty falling asleep	None	Mild	Moderate	Severe	Very
2. Difficulty maintaining sleep, easy to wake up	None	Mild	Moderate	Severe	Very
3. Waking up too early	None	Mild	Moderate	Severe	Very
4. Your satisfaction with your sleep condition	Very	Satisfied	Somewhat	Dissatisfie	Very
5. How much do you think your sleep problems affect your daily life (for example: level of fatigue during the day, ability to work or do household chores, attention, memory, mood, etc.)	None	Mild	Moderate	Severe	Very
6. Compared to others, how much do you think your sleep problems affect your quality of life	None	Mild	Moderate	Severe	Very
7. How concerned are you about your insomnia	None	Mild	Moderate	Severe	Very
Evaluator:	Total score:				
